# Inhibition of branched-chain alpha-keto acid dehydrogenase kinase augments the sensitivity of ovarian and breast cancer cells to paclitaxel

**DOI:** 10.1038/s41416-022-02095-9

**Published:** 2022-12-16

**Authors:** Suad Lateef Ibrahim, Mohammed Najim Abed, Gehad Mohamed, Joshua C. Price, Marwan Ibrahim Abdullah, Alan Richardson

**Affiliations:** 1 The School of Pharmacy and Bioengineering, Guy Hilton Research Centre, Keele University, Thornburrow Drive, Stoke-on-Trent, Staffordshire ST4 7QB UK; 2 Present Address: Clinical and Laboratory Science, Faculty of Pharmacy, University of Kufa, Kufa, Iraq; 3 Present Address: University of Mosul, College of Pharmacy, Nineveh Province, Mosul, Iraq; 4 Present Address: Waveoptics, Abingdon, Oxfordshire OX14 4SE UK; 5 Present Address: Al-Salam Teaching Hospital, Mosul, Iraq

**Keywords:** Cancer therapeutic resistance, Cancer metabolism

## Abstract

**Context:**

Many cancer patients who initially respond to chemotherapy eventually develop chemoresistance, and to address this, we previously conducted a RNAi screen to identify genes contributing to resistance. One of the hits from the screen was branched-chain α-keto acid dehydrogenase kinase (BCKDK). BCKDK controls the metabolism of branched-chain amino acids (BCAAs) through phosphorylation and inactivation of the branched-chain α-keto acid dehydrogenase complex (BCKDH), thereby inhibiting catabolism of BCAAs.

**Methods:**

We measured the impact on paclitaxel sensitivity of inhibiting BCKDK in ovarian and breast cancer cell lines.

**Results:**

Inhibition of BCKDK using siRNA or two chemical inhibitors (BCKDKi) was synergistic with paclitaxel in both breast and ovarian cancer cells. BCKDKi reduced levels of BCAA and the addition of exogenous BCAA suppressed this synergy. BCKDKi inactivated the mTORC1-Aurora pathway, allowing cells to overcame M-phase arrest induced by paclitaxel. In some cases, cells almost completed cytokinesis, then reverted to a single cell, resulting in multinucleate cells.

**Conclusion:**

BCKDK is an attractive target to augment the sensitivity of cancer cells to paclitaxel.

## Introduction

Ovarian cancer is the most fatal gynaecologic malignancy and it is responsible for 150,000 deaths per year [1]. Initially, 80% of ovarian cancer cases respond to standard treatment comprising tumour debulking surgery followed by carboplatin/paclitaxel combination therapy. Unfortunately, most patients who initially respond to therapy ultimately relapse with drug-resistant disease. As a result, only 45% of patients survive for 5 years after diagnosis, prompting the search for novel strategies to overcome drug resistance [1]. The outlook for many breast cancer patients is substantially better than this due to the development of effective targeted agents. However, patients with advanced disease generally have a poor prognosis and for those with triple-negative disease, pharmacological treatment options are primarily chemotherapy such as paclitaxel [2].

To identify drug targets that could be used to increase the sensitivity of ovarian cancer cells to carboplatin or paclitaxel, we have previously evaluated genes whose expression was increased in chemoresistant tumours [3] and identified those whose repression by RNAi increased sensitivity to carboplatin or paclitaxel. One of the first genes we identified was autotaxin [3–5] and its inhibition accelerates apoptosis induced by carboplatin [3] and by paclitaxel [6]. Another hit from the screen, CYR61, also contributes to drug resistance [7, 8]. Together, this demonstrates the validity of our strategy. Here, we have analysed a further hit from the screen, branched-chain α-keto acid dehydrogenase kinase (BCKDK) which was implicated in setting sensitivity to paclitaxel.

BCKDK is a mitochondrial enzyme that is part of the branched-chain α-keto acid dehydrogenase complex (BCKDH) that catabolises the branched-chain amino acids (BCAA) leucine, isoleucine and valine [9]. BCAA are deaminated by branched-chain amino acid transferase (BCAT-1/2) and the resulting α-keto acids are decarboxylated by the BCKDH complex. BCAA metabolism can be reprogrammed to support cancer growth. Plasma or tumour levels of BCAA are increased in patients with breast, pancreatic, lung and liver cancers and in some cases, this has been linked to patient survival [10–15]. How the pathway is deregulated appears to vary between tumour types as it may support BCAA production [13, 16, 17] or it may support BCAA uptake and catabolism to provide intermediates for other metabolic pathways [18–20]. Stromal cells may also be reprogrammed to produce BCAA pathway intermediates to support tumour cells [21]. Many enzymes involved in BCAA metabolism are deregulated in cancer. The expression of BCAT-1 is increased in glioblastoma, leukaemia, liver, breast, and ovarian cancer and its knockdown represses growth [22–25]. BCAT-2 expression is increased in several other cancer types. BCAA metabolic enzymes are also the target of oncogenes. For example, BCAT-1 is regulated by Myc, K-Ras as well as by HIF and BCKDK is phosphorylated by Src [26]. BCKDK expression is increased in high-grade ovarian cancer [27] and in chemotherapy-resistant disease [28]. In colorectal and non-small cell lung cancer, expression of BCKDK correlates with poor patient survival [12, 29]. Apart from BCAA metabolism, BCKDK also regulates other cancer-relevant pathways through the direct phosphorylation of MEK [29] and ATP citrate lyase [30]. Inhibition of BCKDK reduces the growth of both ovarian and colorectal cancer cells and xenografts [27, 29]. Here we show that inhibition of BCKDK using either siRNA or the chemical inhibitors 3,6-dichlorobenzothiophene-2-carboxylic acid (BT2/DCBC) or (S)-2-chloro-4-methylvaleric acid (CMVA) increases the sensitivity of ovarian and breast cancer cells to paclitaxel.

## Materials and methods

### Cell culture and chemical agents

Cell lines were obtained from either ATCC, ECCAC, NIH-NCI or JCRB and were used at low passage number and routinely tested for mycoplasma. COV-318, COV-362, Igrov-1 MDA-MB-231, MDA-MB-468 and MCF-7 cells were grown in DMEM. Normal human ovarian epithelial cells (HOE), OVCAR-4, OVCAR-3, OVCAR-8 and OVSAHO cells were grown in RPMI, and FUOV-1 cells were grown in a 1:1 DMEM/F-12 nutrient mixture. HAP-1 cells and BCKDK knockout cells (Horizon discovery) were grown in Iscove’s Modified Dulbecco’s Medium (IMDM). Media were supplemented with L-Glutamine (2 mM), 10% FCS and antibiotic (penicillin–streptomycin; 50 IU/mL). In addition, the growth media for OVCAR-3 cells was further supplemented with 0.11 g/L sodium pyruvate and 0.01 mg/mL Insulin.

Drugs were prepared in dimethylsulfoxide (DMSO): 5 mmol/L paclitaxel (Sigma-Aldrich); 50 mmol/L CMVA (Tokyo Chemical Industry) and 50 mmol/L DCBC (Sigma-Aldrich).

### Preparation of bespoke media with different concentrations of BCAA

Earle’s Balanced Salt Solution (EBSS; Gibco #240010) was supplemented with 0.4 mM L-serine, 0.2 mM L-methionine, 0.2 mM L-histidine monohydrochloride, 0.8 mM L-threonine, 0.399 mM L-arginine monohydrochloride, 0.8 mM L-lysine monohydrochloride, 0.4 mM L-phenylalanine, 0.2 mM L-cystine hydrochloride, 0.4 mM glycine, 0.078 mM L-tryptophan, 0.248 µM ferric nitrate nonahydrate and filtered. The solution was further supplemented with 1× MEM vitamins solution (Gibco, containing calcium pantothenate, choline chloride, folic acid, inositol, niacinamide, pyridoxine HCl, riboflavin, thiamine HCl), 1 mM sodium pyruvate, 2 mM L-Glutamine, 10% dialysed foetal bovine serum (Gibco), and 50 IU/mL penicillin/streptomycin. A “100×” stock of BCAA (Sigma-Aldrich) was prepared containing 79.9 mM leucine, isoleucine and valine and used to supplement the media as required.

### Cell growth assays

Cell growth assays were conducted and analysed as previously described [31]. In the drug combination studies, a range of concentrations of paclitaxel were tested with 0, 30 or 100 μM fixed concentrations of the BCKDK inhibitors (CMVA and DCBD).

Spheroid culture was performed by using GravityTRAP™ (Tissue Re-aggregation and Assay Plate) Ultra-Low Attachment (ULA) plate (InSphero) according to the manufacturer’s instructions. Cell suspensions (250 cells/70 µL) were dispensed to the bottom of the plate’s well and briefly centrifuged.

### Small interfering RNA (siRNA) transfection and Q-PCR

Cells were transfected with 100 nM of BCKDK siRNA (Dharmacon) in the presence of 0.1% DharmaFECT 1 as previously described [3]. The siRNAs were:

BCKDK#1 GAAAGGAGCAAGACGAUGA

BCKDK#2 GCACGUGCAUGAGCUAUAU

BCKDK#3 GCAGAGGGCCUACGUGAGA

BCKDK#4 GUACUCGUCUCUCACCAAA

BCKDK#17 UGGCAGAGGGCCUACGUGA

Non-targeting siRNA #1 (NT#1; Dharmacon) was also included as a control.

To measure knockdown, RNA was isolated from transfected cells using RNeasy Plus Mini Kit (QIAGEN), cDNA was prepared from RNA by using Transcriptor reverse transcriptase (Roche) and anchored oligo-dT (Invitrogen). BCKDK was measured by Q-PCR (SybrGreen, Abgene) with the primers AATGGCGGTGGACTGTTC and AGGATGCTGACAGGCTCA and normalised to β-actin.

### Measurement of cell viability and apoptosis

Trypan blue staining was used to quantify the viability of cells by seeding 0.5 mL medium containing 10^5^ cells in each well of 12-well plates. The next day, cells were exposed to the indicated concentrations of chemical agents for 48 h (HAP-1 cells were analysed after 24 h) and stained with 0.2% trypan blue.

The expected effect of drug combinations was calculated by using the Bliss independence criterion:

$${{E}_{({x},{y})}} = {{E}_{({x})}} + {{E}_{({y})}} - {{E}_{({x})}}^{*} {{E}_{({y})}}$$

*E*_(*x*,*y*)_ represents the expected effect obtained from the combination, while *E*_(*x*)_ and *E*_(*y*)_ are the effect of each individual drug.

To assess apoptosis, cells were seeded in 96-well plates as described for the cell growth assays and exposed to the indicated concentration of paclitaxel and/or BCKDKi. After 48 h, caspase activity was measured with the Caspase 3/7 Glo-reagent (Promega).

For determination of PARP cleavage, cells were seeded and treated in the same way described for trypan blue assay and lysed with RIPA buffer as described [32]. Lysates were analysed on 4–20% Tris-Glycine Nusep gel, and immunoblotting was performed using an anti-PARP antibody (antibodies used in this study are listed in Table S1).

### Cell synchronisation

In all, 10^5^ MCF-7 or OVCAR4 were seeded per well in 6-well plates. MCF-7 cells were treated with CMVA and DCBC (100 μM) and after 24 h 1 μg/mL nocodazole (Sigma Aldrich) was added. After a further 24 h, the cells were released from block by washing three times with PBS and 500 μL of fresh growth medium containing CMVA/DCBC (100 μM) was added. Samples were analysed after a further 24 h. Alternatively, OVCAR4 cells were treated with nocodazole for 24 h and then the media supplemented with CMVA/DCBC (100 μM). After a further 24 h, the nocodazole was removed by washing with PBS and media replaced with fresh media containing CMVA/DCBC (100 μM).

### Intracellular BCAA concentrations

In all, 4 × 10^4^ OVCAR-4 or 2 × 10^4^ MCF-7 cells were seeded per well of 96-well plates. After 24 h, cells were exposed to 100 μM of CMVA and DCBC for 48 h. The cells were washed several times with PBS and intracellular BCAA measured using a commercial kit (Promega) and normalised to total protein.

### Video microscopy

MCF-7 or MDA-MB-468 (80 µL of 50,000 cells/mL) were seeded in 96-well plates. The next day, the culture medium was removed, and replaced with 80 µL media containing CMVA (100 µM), or DCBC (100 µM). After another 48 h paclitaxel (9.5 nM, equivalent to IC_50_ in cell growth assays) was added and 4 images per well were collected every 17 min for another 24 h (CELLCYTE 10X, Cellink).

### Flow cytometry

Cells were treated with 9.5 nM paclitaxel and/or 100 µM CMVA/DCBC for 48 h. Non-attached cells were collected and combined with the attached cells that were collected by trypsinisation. After washing in PBS and centrifugation, the cells were fixed and stained with 0.05 mg/mL propidium iodide.

## Results

**Fig. 1:**
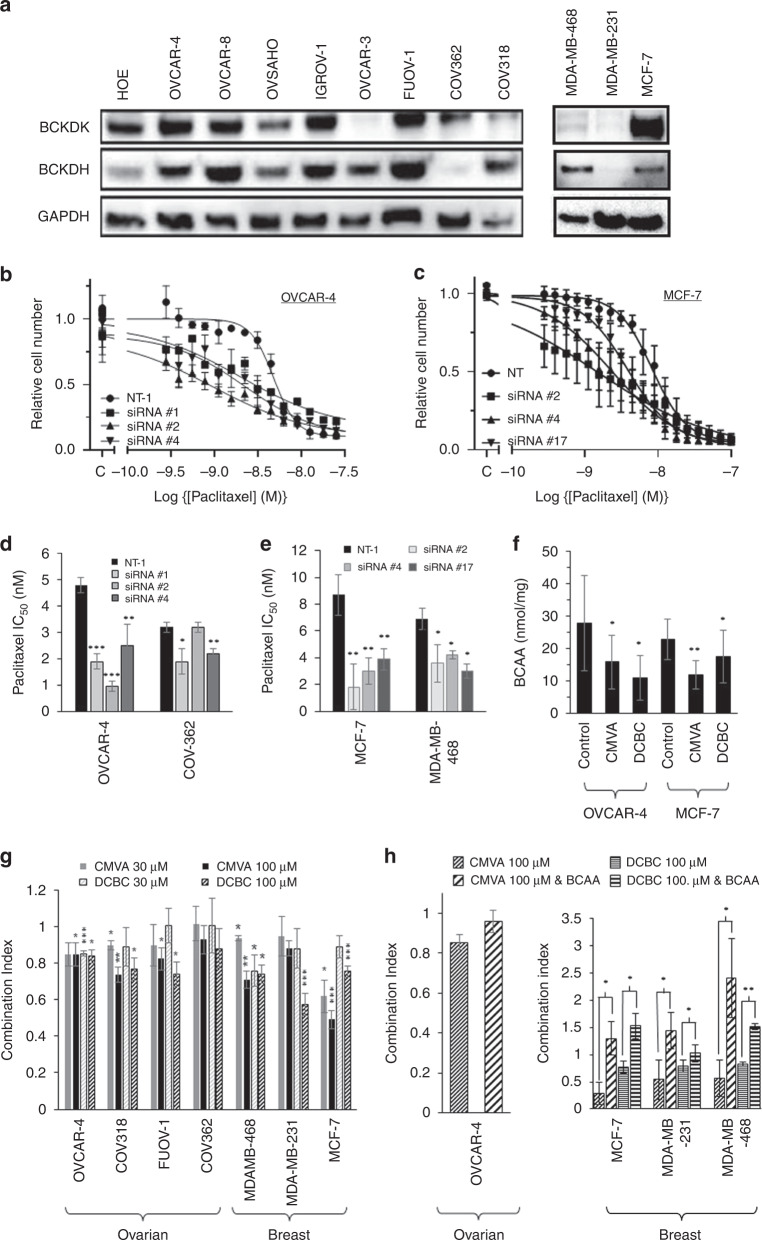
Expression of BCKDK and BCKDH in different cell lines and effect of knockdown on paclitaxel sensitivity. **a** The expression of BCKDK and BCKDH were determined by western blotting of protein lysates from the indicated cell lines. The results shown are representative of the three experiments and were also quantified (Fig. S1). **b**, **c** The sensitivity of OVCAR-4 (**b**) or MCF-7 (**c**) cells to paclitaxel was measured in cell growth assays after knockdown of BCKDK with 3 separate siRNA. The relative cell number was determined by staining with SRB and the results (mean ± S.D., *n* = 3) were normalised to the absorbance measured in the absence of paclitaxel (labelled “C” on the axis). **d**, **e** The potency of paclitaxel in cell growth assays using the indicated ovarian (**d**) or breast cancer cells (**e**) after knockdown of BCKDK with siRNA was quantified. The results (mean IC_50_ ± S.D., *n* = 3) are significantly different from those measured in cells transfected with a non-targeting siRNA (NT-1; ***P < 0.001; **P < 0.01; *P < 0.05; paired *t* test) where indicated. **f** BCAA were measured in either OVCAR-4 or MCF-7 cells exposed to 100 µM CMVA or DCBC for 48 h and intracellular BCAA measured. The results (mean ± SD, *n* = 3) were normalised to total protein per well and are significantly different to that measured in cells treated with the drug vehicle as indicated (*P < 0.05; **P < 0.01 paired *t* test). **g** Combinations of paclitaxel and the indicated concentrations of either CMVA or DCBC (which on their own inhibited cell growth by <5%) were assessed in cell growth assays and the combination index determined. The results (mean ± S.D., *n* = 3) were significantly different from unity were indicated (***P < 0.001; **P < 0.01; *P < 0.05; paired *t* test). **h** Combinations of paclitaxel and either CMVA or DCBC (both 100 µM) in the presence or absence of exogenous BCAA (1 mM) were assessed in cell growth assay. The combination index (mean ± S.D., *n* = 3) were significantly altered by the inclusion of exogenous BCAA where indicated (***P < 0.001; **P < 0.01; *P < 0.05; paired *t* test).

To confirm the results of our screen that identified BCKDK as a potential determinant of the sensitivity of ovarian cancer cells to paclitaxel [3], we first assessed the expression of BCKDK and BCKDH in eight ovarian cancer cell lines including those that have been shown to be among those most representative of high-grade serous ovarian cancer [33]. Considering that paclitaxel is also used in the treatment of breast cancer, we also analysed BCKDK expression in three breast cancer cell lines. In the ovarian cancer cells, immunoblotting demonstrated that BCKDK was highly expressed in COV-362, FUOV-1, IGROV-1 and OVCAR-8 and to lesser extent in COV-318 and OVCAR-3 cell lines. BCKDH was detected in all cell lines, but it was most abundant in COV-318, OVCAR-4 and FUOV-1 with notably poor expression in COV-362 cells (Fig. 1a, quantified in Fig. S1A, B). In the breast cancer cells, BCKDK was abundant in MCF-7 cells, weakly detected in MDA-MB-468 cells and almost undetectable in MDA-MB-231 cells. BCKDH was also not readily detectable in the MDA-MB-231 cells.

In our previous screen, only conducted once using pooled siRNA and two drug concentrations, knockdown of BCKDK increased paclitaxel sensitivity [3]. To validate the results, we knocked-down the expression of BCKDK using different individual siRNA and we used cell growth assays to measure the impact on the sensitivity to a broad range of concentrations of paclitaxel. The siRNA reduced the level of BCKDK in ovarian and breast cancer cells (Fig. S2). Consistent with the results of the screen, knockdown of BCKDK with three separate siRNA increased the sensitivity of OVCAR-4 to paclitaxel (Fig. 1b, d) compared to cells transfected with a non-targeting siRNA, NT-1. Less striking effects were observed in COV-362 cells which expressed relatively little BCKDH (Fig. 1d). In the breast cancer cells, knockdown of BCKDK with three separate siRNA also increased the sensitivity of MCF-7 and MDA-MB-468 breast cancer cells to paclitaxel (Fig. 1c, e).

We next evaluated whether the previously described inhibitors of BCKDK (BCKDKi), CMVA [34] and DCBC [35] also increased sensitivity to paclitaxel. Inhibition of BCKDK is anticipated to relieve the inhibition of BCKDH by BCKDK and consequently promote BCAA metabolism. As expected, both inhibitors reduced intracellular BCAA levels in OVCAR-4 ovarian and MCF-7 breast cancer cells (Fig. 1f). To evaluate synergy between these inhibitors and paclitaxel, we used OVCAR-4, COV-362, COV-318 and FUOV-1 ovarian cancer cells which showed a range of expression levels of BCKDK and BCKDH as well as the three breast cancer cell lines. Before performing drug combinations studies, we established that both drugs when used on their own had minimal effect in cell growth assays at concentrations up to 100 µM (Table S2). Drug combination studies were consequently performed combining either 0, 30 or 100 μM fixed concentrations of each BCKDKi with a range of concentrations of paclitaxel. In cell growth assays, both CMVA and BCKDK were synergistic (combination index, C.I. < 1.0) with paclitaxel using OVCAR-4, COV-318 and FUOV-1 ovarian cancer cells and also using the breast cancer cell lines MDA-MB-468 and particularly MCF-7 (Fig. 1g). Notably, synergy was less apparent in COV-362 and MDA-MB-231 cells which express relatively low levels of BCKDH. However, the high concentrations of BCKDKi we used, necessitated by their modest potency, raised concerns that they might have “off-target effects”. Considering that these inhibitors reduced intracellular BCAA levels, if the drugs were synergistic with paclitaxel because of an “on-target” effect on BCKDK, supplementation of cell culture media with BCAA would be anticipated to reduce the synergy with paclitaxel, but this would not be expected to be the case if the drugs worked through an “off-target” mechanism. In OVCAR-4 cells, supplementation of the media with the BCAAs (1 mM, which on its own had no effect on cell growth) appeared to reduce synergy between paclitaxel and CMVA, although this did not reach statistical significance, possibly because of the modest synergy seen in cell growth assays with this cell line (Fig. 1h) and the poor potency of CMVA [35]. However, addition of BCAA decreased the synergy observed between both CMVA and DCBC in breast (Fig. 1h) cancer cells, consistent with the BCKDKi working synergistically with paclitaxel through inhibition of BCKDK.

**Fig. 2:**
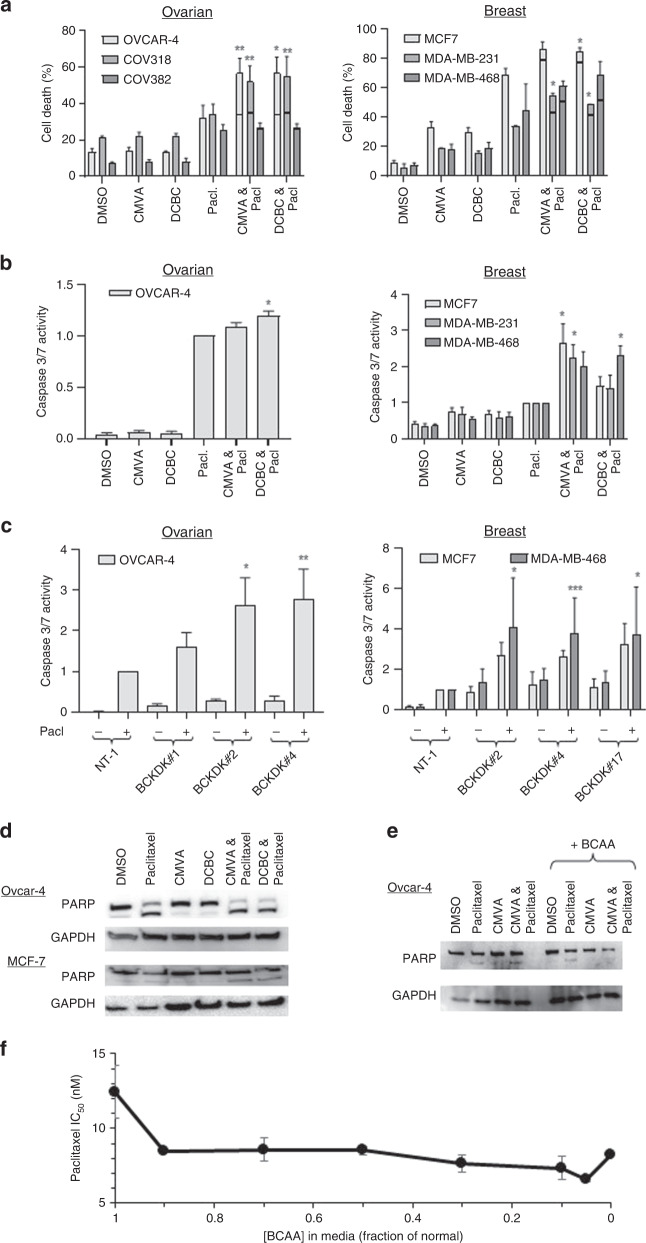
The effect of drug combinations on cell viability, caspase 3/7 activity and PARP cleavage. **a** Ovarian or breast cancer cells were exposed to paclitaxel (9.5 nM), CMVA (100 μM), DCBC (100 μM) or combinations of these drugs as indicated for 48 h and cell viability was assessed by staining with trypan blue. The effect of the drug combination if the results were additive was estimated using the Bliss independence criterion which is indicted with the horizontal bar. The effect of the combination (mean ± S.D., *n* = 3) was significantly different from the expected additive effect where shown (*P < 0.05; **P < 0.005; 1; paired *t* test). **b** Ovarian or breast cancer cells were exposed to the indicated drugs as described in **a** and caspase-3/7 activity determined after 48 h. To control for effects of the drug on proliferation, parallel samples were also exposed to the same drug combinations and relative cell number estimated by staining with SRB. The caspase-3/7 activity was then normalised to relative cell number. The results are expressed as a fraction of the caspase activity measured in cells exposed to paclitaxel alone (mean ± SD, *n* = 3) and are significantly different to those obtained with cells exposed to paclitaxel alone where indicated (*P < 0.05 paired *t* test). **c** Expression of BCKDK was repressed with the indicated siRNA and the cells treated with paclitaxel. After 48 h, caspase-3/7 activity was assessed. The results were normalised to total protein and expressed (mean ± SD, *n* = 3) as proportion of that measured in cells transfected with a non-targetting siRNA (NT-1). The results are significantly different to the caspase activity measured in cells transfected with NT-1 and treated with paclitaxel where shown (*P < 0.05; **P < 0.01; ***P < 0.005; paired *t* test). **d** OVCAR-4 and MCF-7 cells were exposed to the indicated drugs as described in **a** and PARP cleavage was assessed by western blotting. Glyceraldehyde 3-phosphate dehydrogenase (GAPDH) was used as a loading control. The result is representative of three independent experiments. **e** OVCAR-4 cells were exposed to combinations of paclitaxel as described above, in the absence or presence of additional BCAA (1 mM) and PARP cleavage assessed. The result is representative of three independent experiments. **f** MCF-7 cells were grown in the bespoke media prepared to contain BCAA at a fraction of that normally found in DMEM (0.8 mM). The potency of paclitaxel (IC_50_ ± S.D., *n* = 3) was determined in cell growth assays and was significantly different from that measured in the normal medium.

To confirm the synergy, alternative measurements of the activity of the drug combination were used. Consistent with cell growth assays results, the combinations of either DCBC or CMVA with paclitaxel resulted in significantly more cell death in OVCAR-4 and COV-318 ovarian cancer cells and MCF-7 and MDA-MB-468 breast cancer cells than would have been expected from the Bliss independence criterion if the effects of the drugs were additive (Fig. 2a). In contrast, in COV-362 cells and MDA-MB-231 cells, reminiscent of the results obtained in cell growth assays, the effect of the combination was not significantly different from the effect anticipated if the drugs acted in an additive fashion. Notably, these cells expressed low levels of BCKDH (Fig. 1a). The effect of inhibiting BCKDK on paclitaxel induced-apoptosis was investigated by measuring caspase-3/7 activity and PARP cleavage. In OVCAR-4 cells both CMVA and DCBC failed to elicit significant activation of caspase-3/7. However, when cells were exposed to paclitaxel and either CMVA or DCBC, caspase-3/7 activity was significantly more than that observed in cells treated with paclitaxel alone (Fig. 2b), consistent with synergy. In the breast cancer cells, similar results were observed. These results were confirmed by using RNAi in place of the inhibitors. When BCKDK was repressed using 3 different siRNA, no effect on caspase-3/7 activity was observed in OVCAR-4, MCF-7 or MDA-MB-468 cells. However, in both breast and ovarian cancer cells, knockdown of BCKDK with each of the siRNA increased the caspase-3/7 activity induced by paclitaxel compared to cells transfected with a non-targetting siRNA (Fig. 2c). The BCKDKi on their own also had no effect on PARP cleavage in OVCAR-4 ovarian and MCF-7 breast cancer cells. However, both CMVA and DCBC potentiated the PARP cleavage induced by paclitaxel (Fig. 2d), again confirming synergy between paclitaxel and the BCKDK inhibitors. Supplementation with BCAA prevented the potentiation of PARP cleavage by BCKDKi (Fig. 2e).

Considering that the BCKDKi reduced BCAA levels, we prepared bespoke media with different concentrations of BCAA, thereby mimicking the effect of the inhibitors. As anticipated from the foregoing experiments, reducing BCAA in the culture media increased the potency of paclitaxel (Fig. 2f).

**Fig. 3:**
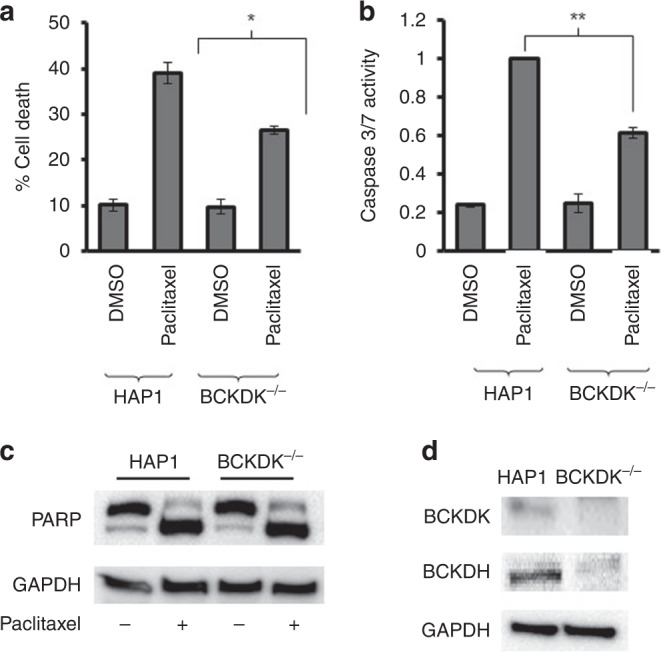
The effect of paclitaxel in cells edited to lack BCKDK. **a** HAP-1 and BCKDK^−/−^ cells were treated with paclitaxel (5 nM, 24 h) and the viable cell number was determined by staining with trypan blue. The number of dead cells (mean ± S.D., *n* = 3) was significantly less (*P < 0.05, paired *t* test) in the BCKDK^−/−^ cells compared to the parental HAP-1 cells **b** Caspase-3/7 activity was determined in HAP1 and BCKDK^−/−^ cells following exposure to paclitaxel (5 nM, 24 h). The activity of caspase was normalised to relative cell number determined by SRB staining of a comparable experiment performed at the same time to control for any effect of the drug on cell number. The results are expressed as a fraction of the caspase-3/7 activity measured in the HAP1 cells (mean ± S.D., *n* = 3) and are significantly different where indicated (**P < 0.095, paired *t* test). **c** The indicated cells were exposed to paclitaxel (5 nM, 24 h) and PARP cleavage was determined by western blotting. GAPDH was used as a loading control. The results are representative of three experiments. **d** BCKDK and BCKDH were measured by immunoblotting of lysates prepared from untreated HAP-1 and BCKDK^−/−^. GAPDH was used as a loading control. The image is representative of three experiments.

The foregoing results led us to hypothesise that cells lacking BCKDK would be more sensitive to paclitaxel than cells expressing BCKDK. We compared the potency of paclitaxel in HAP-1 haploid cells with corresponding cells that had been edited to knock-out the remaining BCKDK allele. Unexpectedly, the BCKDK^−/−^ cells did not show a significant difference in sensitivity to paclitaxel in cell growth assays and if anything were mildly less sensitive to paclitaxel (IC_50_ = 4.8 ± 1.1 nM) than parental cells (IC_50_ = 3.9 ± 1.1 nM). To explore this further, the activity of paclitaxel in HAP-1 and BCKDK^−/−^ cells was also evaluated by cell viability, caspase-3/7 activity and PARP cleavage assays. After 24 h of treatment with paclitaxel, there was significantly more cell death in parental HAP-1 cells compared to the BCKDK^−/−^ cells (Fig. 3a), again the opposite of the result predicted by our hypothesis. The caspase-3/7 activity induced by paclitaxel was also more pronounced in HAP-1 parental cells than in the BCKDK^−/−^ cells (Fig. 3b). Similarly, paclitaxel-induced PARP cleavage was more evident in HAP-1 cells compared to BCKDK^−/−^ cells (Fig. 3c). Even in 3D culture, BCKDK^−/−^ cells were less sensitive to paclitaxel than HAP-1 cells (Fig. S3). Thus, the results obtained in four different assays were consistent with the knockout cells being less sensitive to paclitaxel, in apparent contradiction to the previous results. To resolve this discrepancy, we measured BCKDK by immunoblotting in the untreated HAP-1 and BCKDK^−/−^ cells. As expected, BCKDK was not detectable in BCKDK^−/−^ cells but was detectable in the parental line. However, in the BCKDK^−/−^ cells, the level of BCKDH, the substrate of BCKDK, was also markedly reduced (Fig. 3d), consistent with adaptive down-regulation of BCKDH.

**Fig. 4:**
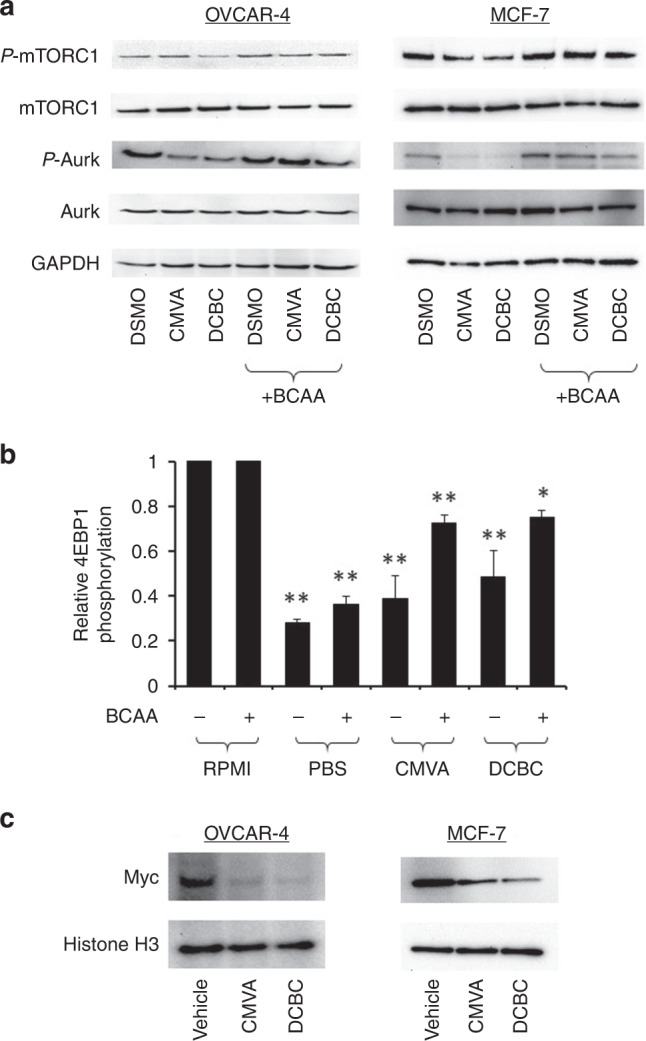
Inhibition of the mTORC1-Aurora pathway by BCKDKi is abrogated by inclusion of exogenous BCAA. **a** OVCAR-4 or MCF-7 cells were synchronised with nocodazole as described in the methods, treated with DMSO or 100 μM CMVA and DCBC for 72 h, in the absence or presence of additional (1 mM) BCAA, lysed and analysed by immunoblotting as shown. The results are representative of three separate experiments and the quantified images are shown in Fig. S5. **b** Phosphorylation of 4E-BP1 (Thr^37/46^) was analysed by a ELISA in MCF-7 and cells. The indicated cells were treated with either drug solvent (control) or 100 μM of CMVA or DCBC for 72 h or the media replaced with PBS 1 h prior to the ELISA. The phosphorylation of 4EBP-1 (mean ± S.D., *n* = 3, expressed as a fraction of that measured in cells exposed to solvent) was significantly different where indicated (one-way ANOVA with Tukey’s Multiple Comparison Test; *P < 0.01; **P < 0.001) to that measured in cells exposed to solvent. **c** OVCAR-4 or MCF-7 cells were treated with the BCKDK inhibitors as described in **a** and Myc and H3 measured by immunoblotting (image representative of three independent experiments).

We next explored the mechanism of the synergy between BCKDK inhibition and paclitaxel. We noted a striking report by Platani and co-workers [36] which demonstrated that the SEACAT/GATOR2 complex, which is regulated by levels of BCAA and which controls mTORC1, regulates the mitotic kinases Aurora and Plk1. Aurora kinases play roles in the initiation, progression and exit from M-phase. Considering that paclitaxel disrupts mitotic spindle function and activates the spindle assembly checkpoint, we hypothesised that BCKDKi reduce BCAA level, mTORC1 and Aurora activity and consequently are synergistic with paclitaxel by further disrupting proper mitotic spindle function during mitosis. Phosphorylation of Aurora peaks during M-phase. To assess the effect of BCKDKi on Aurora phosphorylation, we synchronised MCF-7 or OVCAR-4 cells with nocodazole, released the cells by washing and measured the effect of CMVA or DCBC at the time when Aurora phosphorylation peaked in untreated cells (Fig. S4A). We co-ordinated this with the time required for BCKDKi to reduce BCAA levels (the experimental design is shown in Fig. S4B). Cells exposed to either CMVA or DCBC showed reduced phosphorylation of mTORC1 and Aurora (Fig. 4a, quantified in Fig. S5). This was blocked by supplementation of the media with BCAA, demonstrating the effect of the inhibitors depended on BCAA. Phosphorylation of the mTORC substrate 4EBP1 measured in an ELISA was also reduced by BCKDKi and this was again ameliorated by supplementing the media with BCAA (Fig. 4b). Translation of Myc is controlled by mTORC1 and Myc is also stabilised by binding to Aurora. CMVA and DCBC were both able to reduce levels of Myc assessed by immunoblotting of lysates from OVCAR-4 and MCF-7 cells (Fig. 4c, quantified in Fig. S6).

**Fig. 5:**
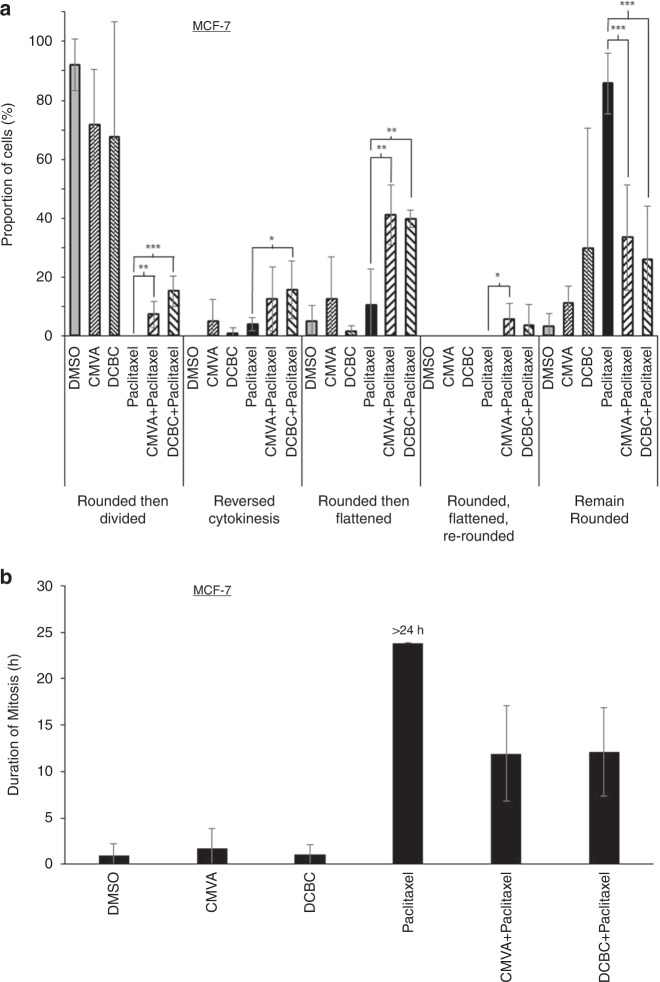
Effect of BCKDK inhibitors on M-phase arrest induced by paclitaxel. **a** MCF-7 cells were exposed to the indicated combinations of paclitaxel (9.5 nM), CMVA (100 µM) or DCBC (100 µM) and monitored by time-lapse microscopy. The time at which the cells rounded and their subsequent behaviour was classified (mean ± S.D., *n* = 3 total of 100 cells counted for each condition, taken from three separate experiments) as “reversed cytokinesis” (cells started to divide, but then aborted division), “rounded then flattened” (cells did not divide but instead reverted to a flat morphology) or “rounded, flattened then re-rounded (as the previous group, but subsequently rounded again) or “remained rounded” (the cells remained rounded until the end of the recording period). The results were significantly different where shown (ANOVA, *P < 0.05; **P < 0.01; ***P < 0.001). A similar analysis was conducted with MDA-MB-468 cells (Fig. S7). The cell fate maps for each of these experimental conditions are presented in Figs. S8–21. **b** The time at which cells rounded was noted and time to flatten again determined and the interval this encompassed taken to represent the time required to complete mitosis. Cells treated with paclitaxel remained rounded throughout the 24 h duration of the video and hence are labelled “>24 h”.

We next considered how inhibition of Aurora and microtubule function might synergistically cause cell death. Aurora inhibitors have previously been reported to cause mitotic slippage, a process by which cells escape M-phase arrest and the spindle assembly checkpoint induced by paclitaxel, consequently dividing prior to complete chromosomal segregation and resulting in multinuclear cells [37]. To assess whether BCKDKi did the same, time-lapse microscopy was used to monitor cell rounding, division and subsequently flattening of the cells in culture; in these experiments, MCF-7 and MDA-MB-468 cells were used because their flat morphology during interphase allowed cell rounding during mitosis to be clearly observed and because OVCAR-4 cells proliferate slowly. The cells were exposed to the drugs for 48 h and then monitored by video microscopy for the subsequent 24 h. During these 24 h almost all of the cells treated with drug solvent rounded, divided and became flat again (Fig. 5a; see Figs. S8–21 for a fate map of each cell analysed and supplementary videos for examples). Cells treated with paclitaxel rounded and remained so for the entire period of recording. However, the prior addition of either CMVA or DCBC to cells treated with paclitaxel significantly reduced the number of cells that remained rounded to the end of the recording, and instead after rounding many of the cells then flattened without dividing. Alternatively, after rounding, a small but significant proportion of the cells started cytokinesis, or even almost completely separated, but failed to fully separate and reverted to a single cell (“reversed cytokinesis”, see supplementary videos). In the MDA-MB-468 cells the addition of the BCKDK inhibitors also allowed the cells to escape their rounded morphology, but in this case the cells either divided or flattened again without dividing; reversal of cytokinesis was rarely observed (Fig. S7A). Some cells fragmented, consistent with them undergoing apoptosis (recorded as “cell lysis”). We also quantified the period between cells rounding and either dividing or becoming flat again to estimate the time taken to exit from mitosis. Cells exposed to drug solvent completed mitosis typically within 1 h. No (0/100) MCF-7 cells exposed to paclitaxel were observed to exit mitosis during 24 h of video analysis. However, 62/100 cells exposed to CMVA and paclitaxel, and 68/100 cells exposed to DCBC and paclitaxel, where able to exit mitosis in approximately 12 h (Fig. 5b). Similar results were obtained in MDA-MB-468 cells (Fig. S7B).

**Fig. 6:**
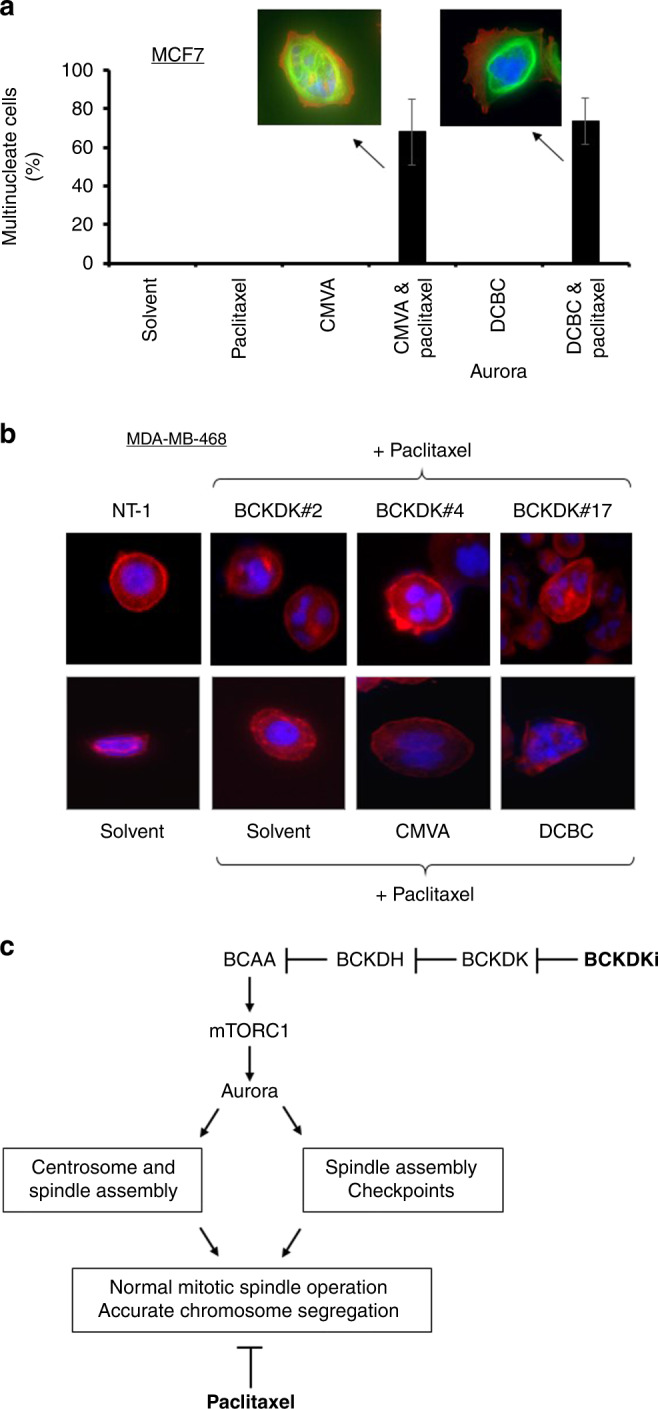
BCKDKi and paclitaxel induce the formation of multinuclear cells. **a** MCF-7 cells were exposed to CMVA or DCBC for 48 h, and subsequently paclitaxel and the BCKDK inhibitors for a further 24 h before fixation and staining with DAPI and anti-actin antibody. The number of multinucleate cells was determined by microscopy (mean ± S.D. *n* = 3, total of >100 cells counted for each condition). **b** MDA-MB-468 cells were exposed to the same combination of drugs or to three separate siRNA to repress the expression of BCKDK. The cells were stained with DAPI (blue) or with an antibody to F-actin (red). The number of multinucleate cells was determined by microscopy. **c** The proposed mechanism for augmentation of paclitaxel activity. Paclitaxel prevents the assembly of the mitotic spindle causing activation of the spindle assembly checkpoint and arrest in M-phase. Inhibition of BCKDK relieves the inhibition of BCKDH by BCKDK, thereby promoting BCAA metabolism. Activation of mTORC1 is dependent upon branched-chain amino acids so their subsequent metabolism by BCKDH leads to inactivation of mTORC1 and its substrate Aurora. This further impairs mitotic spindle assembly and/or prevent proper functioning of the spindle assembly checkpoint. Consequently, the drugs act synergistically to prevent accurate chromosomal segregation, resulting in cell death.

These data suggested that the BCKDKi allowed the cells to escape the spindle assembly checkpoint. Exposure of cells to aurora inhibitors has previously been reported to result in multinuclear cells. Staining MCF-7 cells that had been exposed to both paclitaxel and the BCKDK inhibitors with DAPI revealed a significant proportion of multinuclear cells (Fig. 6a). A large proportion of multinuclear cells were also observed in MDA-MB-486 cells in which BCKDK had been knockdown down and the cells exposed to paclitaxel (Fig. 6b). These multinuclear cells could potentially represent cells in which their normal DNA was present in separate nuclei or cells which had become polyploid as a result of endoreduplication. To distinguish between these two possibilities, we performed flow cytometry analysis of cells stained with propidium iodide (Fig. S22). After exposure to paclitaxel or paclitaxel and the BCKDKi for 48 h, as expected there was a substantial increase in the ratio of cells in G_2_/M compared to G_1_ cells and a large number of sub-G_1_ cells, consistent with cell death. Approximately 10% of the cells became polyploid, but the number of polyploid cells was comparable in samples treated with either paclitaxel alone or paclitaxel and the BCKDKi.

## Discussion

We evaluated a hit from a prior siRNA-based screen that evaluated genes that are over-expressed in chemoresistant ovarian cancer to identify those genes which contribute to resistance to paclitaxel. We confirmed that inhibition of BCKDK with drugs or siRNA, or decreasing levels of BCAA in culture medium, increased the sensitivity of both ovarian and breast cancer cells to paclitaxel. Analysis of the mechanistic basis of the synergy suggested that is due, at least in part, to inhibition of the mTORC-Aurora pathway, converging with paclitaxel to impair accurate mitosis.

The observation that inhibition of BCKDK sensitises cells to paclitaxel was made in several experimental settings including cell growth, cell viability and two assays to measure apoptosis. Synergy was observed using 3 separate siRNA and two chemically distinct inhibitors. Although chemical inhibitors facilitate the exploration of quantitative pharmacodynamic relationships, a concern associated with them is that they may act through an off-target effect. Inhibition of BCKDK relieves the suppression of BCKDH by BCKDK and so promotes BCAA metabolism. Thus, if the inhibitors were synergistic with paclitaxel as a result of inhibition of BCKDK rather than an off-target effect, supplementation of cells with exogenous BCAA would be expected to nullify the synergy. We supplemented culture media with BCAA and this abolished the synergy suggesting an “on-target” mechanism of action. Considering that the BCKDK inhibitors reduced BCAA levels, we prepared bespoke culture media with lower BCAA concentrations and this also increased sensitivity to paclitaxel, providing a direct demonstration (independent of drug addition) that the reduced BCAA levels increase the sensitivity of cancer cells to paclitaxel. Indeed, in these studies, the effect of reduced BCAA was dramatic and a 10% reduction was sufficient to increase sensitivity to paclitaxel.

To explore the link between BCKDK and paclitaxel sensitivity further, we used cells genetically engineered to lack BCKDK. We anticipated that cells lacking BCKDK^−/−^ would be more sensitive to paclitaxel but in several assays, we found the opposite. However, cells lacking BCKDK also expressed less BCKDH. Considering that BCKDK represses BCKDH activity, the loss of BCKDK would be anticipated to lead to unregulated BCKDH activity. We suggest that BCKDK^−/−^ cells undergo compensatory adaptation and down-regulate BCKDH expression to reduce its activity. This is precedented because adaptive responses in the BCAA metabolic pathway in response to changes in BCAA metabolite levels have been described previously [38]. We argue that the fact that apparently adaptive changes in the BCAA pathway altered the cellular sensitivity to paclitaxel, even though this was converse to the response expected, underscores that there is a link between BCAA levels and cellular sensitivity to paclitaxel. Together with the foregoing data, this suggests that BCKDKi may be useful in the treatment of cancer that has become resistant to paclitaxel. Previous work has also implicated BCAT-1 in resistance to cisplatin [39] and inhibition of BCKDK potentiates the cytotoxic activity of doxorubicin [40], supporting this assertion.

We next considered the mechanism underlying the synergy. A striking report by Platani et al. [36] linking BCAA levels to the regulation of mTORC and hence Aurora provided us with a hypothesis because Aurora kinases regulate mitotic microtubules, the very target of paclitaxel. We hypothesised that BCKDKi and paclitaxel synergistically regulate mitotic spindle function. We used nocodazole to synchronise the cells and although it was removed by washing, we acknowledge the possibility that this could introduce experimental bias because this agent, like paclitaxel, also targets microtubules. However, as predicted by the hypothesis, BCKDK inhibitors reduced phosphorylation of mTORC1 and Aurora kinase and other proteins (4EBP1 and MYC) controlled by mTORC1. Again, supplementation of cell culture media with BCAA reversed the inhibition of this pathway. We propose that inhibition of BCKDK increases BCAA metabolism, resulting in inhibition of mTORC1 and hence Aurora kinases and that this is synergistic with paclitaxel because both drugs act to impair accurate mitosis (Fig. 6c). Consistent with this, knockdown of Aurora increases sensitivity to paclitaxel [41] and paclitaxel and Aurora inhibitors are synergistic in breast [42] and ovarian cancer [43] cells. Furthermore, inhibitors of mTORC are synergistic with paclitaxel [44–46]. Video analysis showed that while cells arrested by paclitaxel remain rounded, inclusion of BCKDKi allowed the cells to escape paclitaxel-induced arrest and revert to a flattened state. Although the BCKDKi did not increase ploidy beyond that known to be induced by paclitaxel [47], a significant number of multinucleate cells were observed. Paclitaxel inhibits the dynamic instability of mitotic microtubules, preventing the formation of the spindles that separate sister chromatids during M phase. The spindle assembly checkpoint (SAC) monitors whether all kinetochores on the chromatids are attached to spindle microtubules and separation is delayed until this condition is fulfilled. Paclitaxel activates the SAC because the impaired mitotic spindles result in unattached kinetochores [48]. Aurora A regulates the maturation of centrosomes which anchor the mitotic spindles during mitosis and maintains the SAC [49]. Consequently, inhibition of Aurora A inactivates SAC and allows cells to exit mitosis. Inhibition of aurora A accelerates escape from M-phase arrest induced by paclitaxel [37]. Aurora B helps ensure the amphitelic attachment of kinetochores to microtubules and facilitates the reattachment of “lagging” chromosomes that have become separated from the mitotic spindle. Inhibition of Aurora B prevents cells from delaying mitosis if chromosomal separation is incomplete [37]. Thus, inhibition of mTORC1 and hence Aurora A or Aurora B by BCKDKi provides a plausible explanation for the synergy between these two agents: paclitaxel induces mitotic arrest due to defective spindle formation and this is augmented by inhibition of Aurora A (Fig. 6c). Alternatively, the imperfect chromosomal segregation induced by paclitaxel renders the cells dependent on activation of Aurora B to prevent exit from M-phase prior to complete chromosomal segregation and BCKDKi prevent this by inhibiting Aurora B [50]. In addition to the effect of BCKDKi on Aurora kinases, inhibition of survival signals from mTORC1 and Akt by increased BCAA metabolism may also contribute to the synergy by disrupting survival signals [51, 52]. Furthermore, BCKDK has been shown to phosphorylate MEK [29] and considering that MEK inhibitors can be synergistic with paclitaxel [53], reduced activity of the Erk pathway caused by BCKDKi may also contribute to the synergy we have observed. Further studies will be necessary to assess the relative contribution of these different pathways.

Here we have suggested that BCKDKi may be useful in the treatment of cancer when combined with paclitaxel. As discussed in the introduction, BCAA metabolism and BCKDK itself are deregulated in cancer and emerging as a potential new target in cancer. Thus, BCKDKi may also be useful as single agents or combined with other drugs targeting metabolism [54, 55]. Some cancers, including ovarian and breast, show alterations in BCAA metabolism that may render them sensitive to BCKDKi. NSCLC use BCAA for protein synthesis or as a source of nitrogen for anabolic reactions [19] and PDAC tumours can become dependent on the provision of BCAA from cancer-associated fibroblasts [21]. Thus, BCKDKi may also be useful to target stromal cells. BCAA are essential amino acids that must be ingested through diet and this may reduce the likelihood of resistance to BCKDKi through the bypass of BCAA deprivation. Additionally, mTORC and Myc are key metabolic regulators, so their inhibition by BCKDKi may have significant metabolic effects that are therapeutically useful. However, numerous issues need to be addressed before BCKDKi can be translated to the clinic. Firstly, substantially improved BCKDKi will be required. BCKDK is an atypical kinase, structurally distinct from the classical kinase family, making this feasible. This will allow confirmation of the observations reported here in animal studies. Secondly, it will be important to identify appropriate biomarkers that can be used to identify patients who are likely to benefit from BCKDKi. The increased expression of BCKDK itself in tumour tissue may suggest the use of BCKDKi, particularly considering BCKDK expression has been correlated with reduced survival of patients with CRC and NSCLC [12, 29]. However, the increase in plasma BCAA that results from either the decreased expression of BCAA metabolic enzymes [13] or the increased expression of BCKDK may offer a less invasive biomarker, potentially of particular use in cancers where increased BCAA levels have been associated with increased cancer risk or reduced survival [11, 13, 14]. Thirdly, considering the apparent adaptation we saw in HAP cells lacking BCKDK, due consideration to the appropriate duration of exposure to BCKDKi will be required, including consideration of continuous or intermittent exposure strategies. Nonetheless, the findings presented here underscore the potential usefulness of targeting BCAA metabolism to treat cancer.

## Supplementary information

Supplementary material

AJ checklist

MCF7 Paclitaxel only

MCF-7 - Rounded cells

MCF-7 - mitosis

MCF-7 Flat rounded Flat

MCF-7 Reverse cytokinesis

MDA-MB-468 paclitaxel only

MDA-MB-468 rounded cells

MDA-MB-468 mitosis

MDA-MB-468 flat round flat

MDA-MB-468 flat round flat 2

MDA-MB-468 reversed cytokinesis

MDA-MB-468 lysis

## Data Availability

Data are available from the authors upon reasonable request.
