# Supplementary material for: Inhibition of branched-chain alpha-keto acid dehydrogenase kinase augments the sensitivity of ovarian and breast cancer cells to paclitaxel
Source: Br J Cancer. 2022 Dec 16;128(5):896–906. doi: 10.1038/s41416-022-02095-9 (PMC9977917; doi:10.1038/s41416-022-02095-9)
Supplement: Supplementary file 1 — Supplementary material [file 41416_2022_2095_MOESM1_ESM.pdf]

Table S1 Antibodies used in this study

Antigen	Antibody
Aurora	Cell Signalling Technology #14475
Phospho-Aurora A T288	Cell Signalling Technology #3079
BCKDK	Bethyl laboratories A303-790A-M
mTORC	Cell Signalling Technology, #2972
Myc	Santa-Cruz, SC-40
Histone H3	Cell Signalling Technology #4499
PARP	Cell Signalling Technology #9542

Table S2: Activity of DCBC and CMVA in cell growth assays.
The potency of CMVA and DCBC as single agents in cell growth assays was determined in the indicate cell lines. Results are expressed as the IC_{50} (mean±S.D,n=3). “>200” indicates the compounds were insufficiently potent for a reliable IC_{50} determination so the IC_{50} is reported as being greater than 200 μM.

Cell line	IC50 (μM)	
	CMVA	DCBC
**OVCAR-4**	>200	>200
**COV362**	>200	>200
**COV318**	>200	>200
**FUOV-1**	>200	>200
**MCF-7**	180 ± 90	120 ± 40
**MDA-MB-468**	>200	120 ± 20
**MDA-MB-231**	>200	>200

S1 Quantification of BCKDK and BCKDH in ovarian and breast cancer cell lines

A

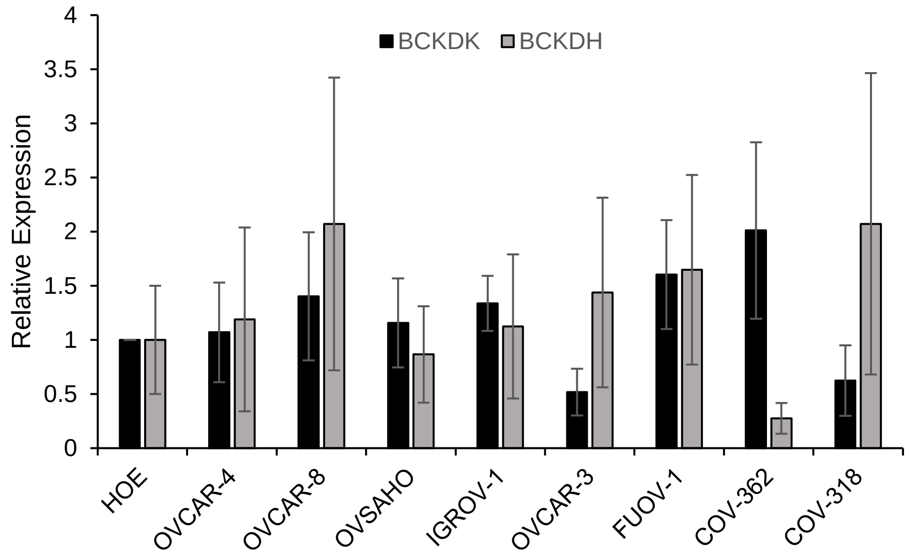

B

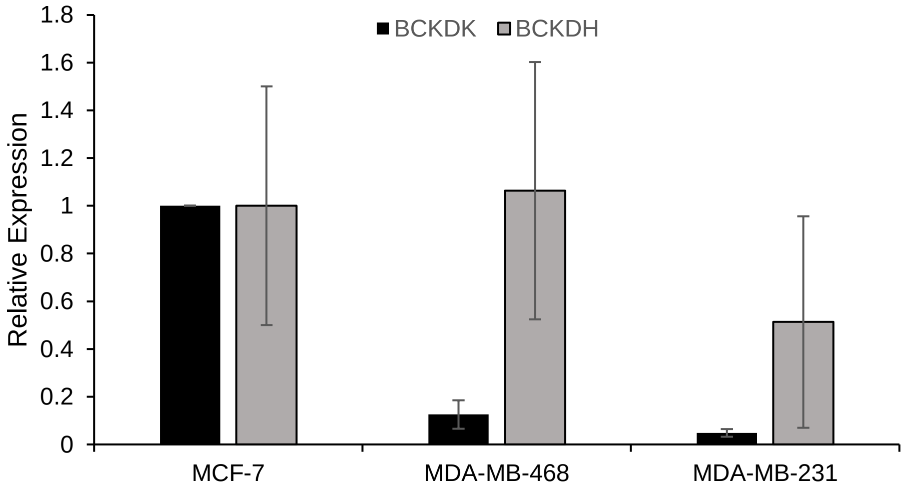

Supplementary figure 1

Quantification of BCKDK and BCKDH expression. The expression of the BCKDK and BCKDH in the indicated ovarian (A) and breast (B) cancer cell lines was analysed by immunoblotting. The resulting chemiluminescent signal was quantified and normalized to GAPDH in each experiment. The results of several experiments (mean ± S.D n= 3) normalized to the expression in HOE (normal human ovarian epithelial cells) or MCF-7 cells respectively.

S2 Confirmation of BCKDK knockdown

A.

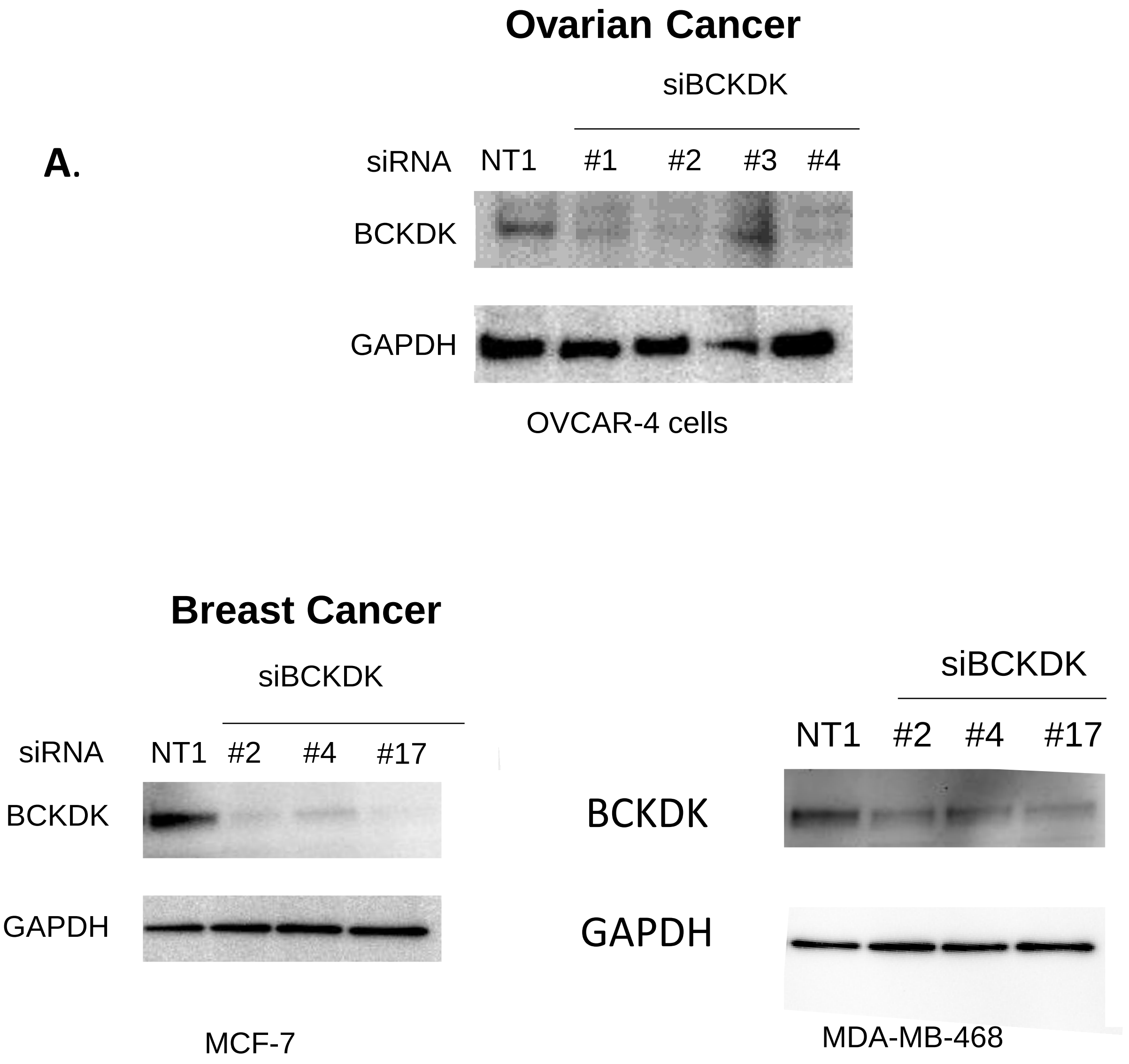

B.

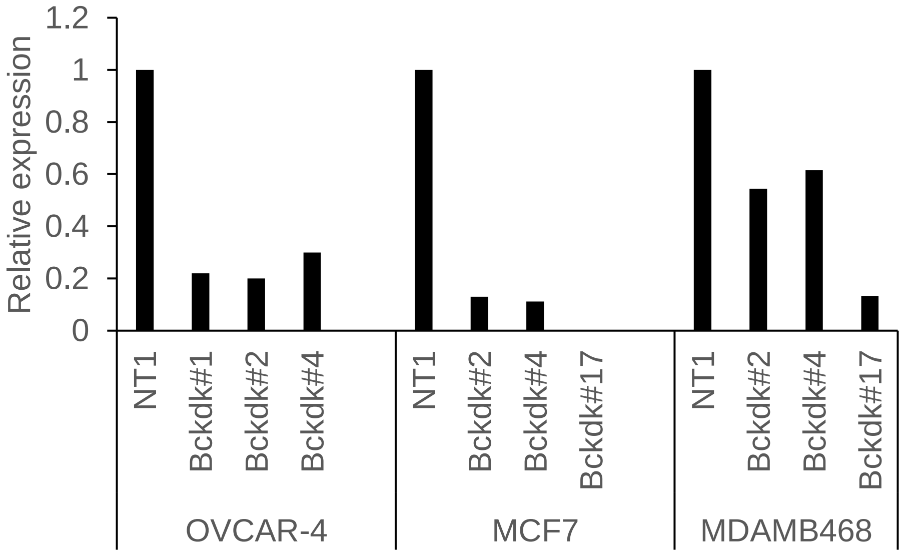

Supplementary figure 2. Repression of BCKDK expression by siRNA. The expression of BCKDK was determined by western blotting of protein lysates obtained from OVCAR-4, MCF-7 or MDA-MB-468 cell lines 48 hours after transfection with the indicated BCKDK siRNA or non-targetting siRNA (NT-1). The results are representative of three experiments. Note that siRNA #1 was replaced with #17 following the supplier's recommendation and that differences in efficiency of knockdown may represent differences in transfection efficiencies between different cell lines. **A**. repression of expression in MDA-MB-468 cells. **B**. Quantification of expression.

S3 Effect of paclitaxel on spheroids on cells lacking BCKDK

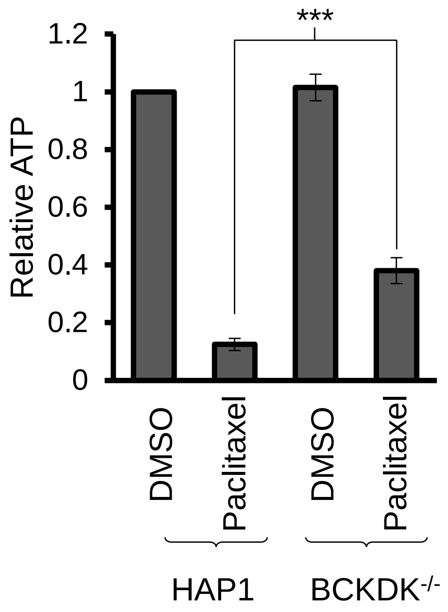

Supplementary figure 3 The effect of paclitaxel (30 nM, 72 hours) was measured using spheroids formed from either HAP-1 or $BCKDK^{-/-}$ cells. Relative viable cell number was assessed by measurement of intracellular ATP and are expressed as a fraction of paclitaxel in HAP-1 cells. The results (mean ± S.D, n=6) are significantly different where indicated (***, $P < 1x\ 10^{-6}$, paired *t*-test).

S4 Experimental design for cell synchronization studies and treatment with BCKDKi

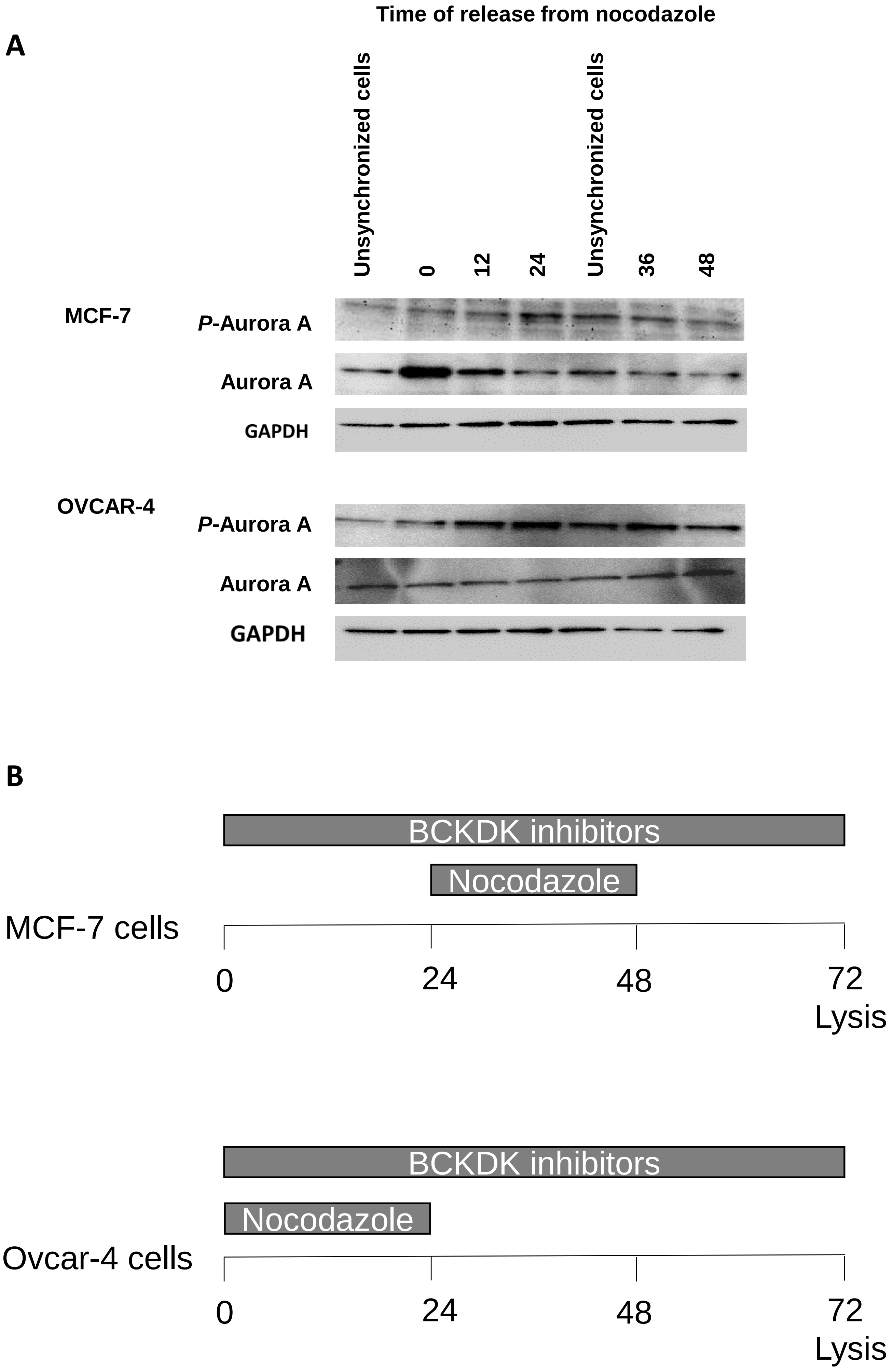

Supplementary figure 4. **A**. The indicated cells were synchronized with nocodazole (1 µg/ml , 24 h), washed and lysed after the indicated times. Aurora phosphorylation was detected by immunoblotting. **B.** Scheme showing the experimental use of nocodazole-induced arrest and BCKDK inhibitors. This scheme was adopted because it allowed both the cells to be synchronized but at the same time allow sufficient time for the BCKDKi to drive BCAA metabolism. Different schemes were used with OVCAR-4 and MCF-7 because of the slower growth of the former cells.

S5 Quantification of immunoblotting to assess the effect of BCKDKi on mTORC and Aurora phosphorylation

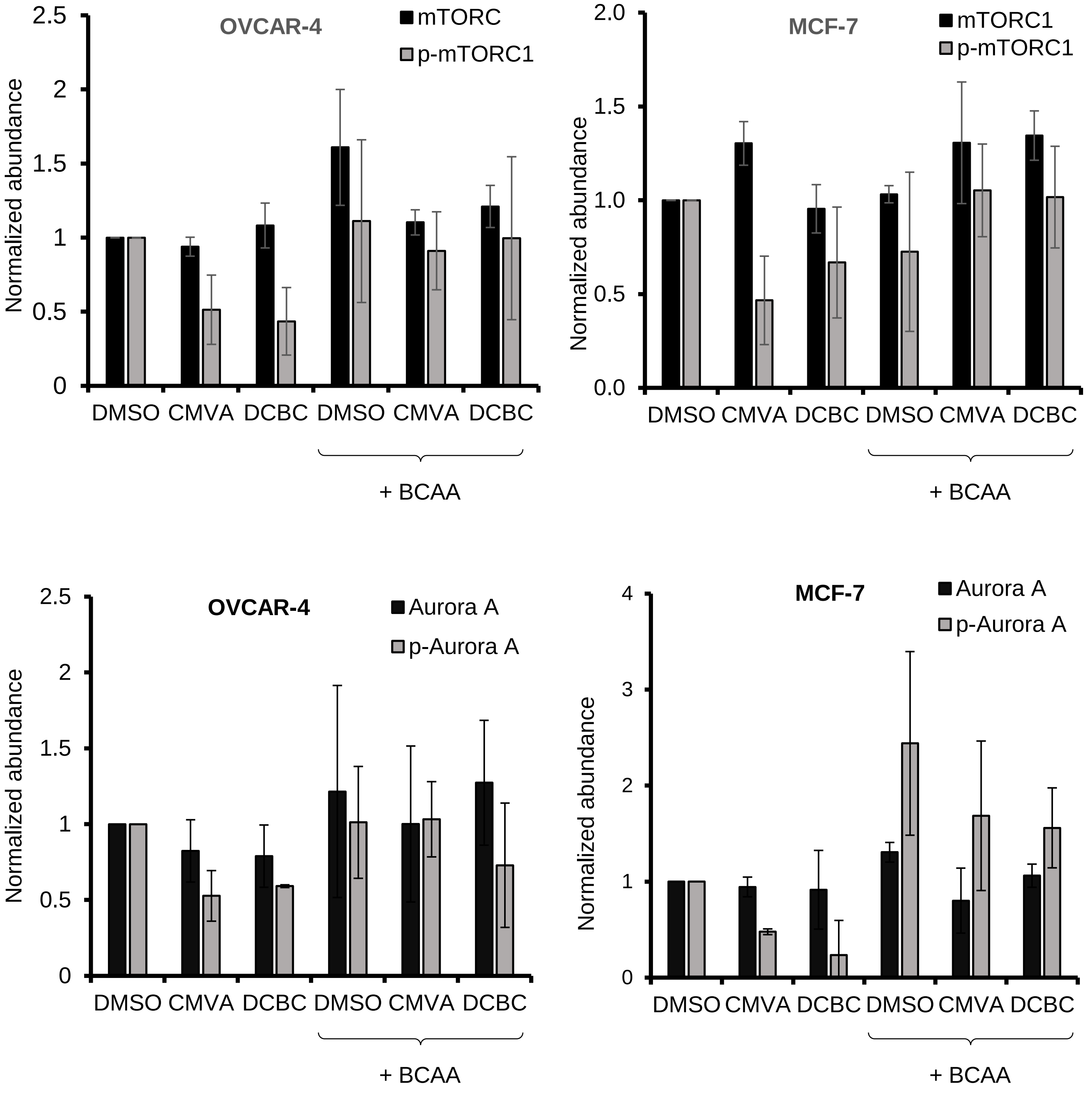

Supplementary Fig 5 Quantification of the effects of BCKDK inhibitors on phosphorylation of mTORC and Aurora. OVCAR-4 or MCF-7 cells were synchronized with nocodazole, then treated with 100 µM CMVA and DCBC for 72h. A second set of cells were supplemented with 1mM BCAAs for 30 min before cell collection. Following immunoblotting, the intensity of the signal was quantified and normalized to the corresponding bands for GAPDH. These results are from three independent experiments and are presented as means ± SD.

S6 Effect of BCKDKi on Myc expression

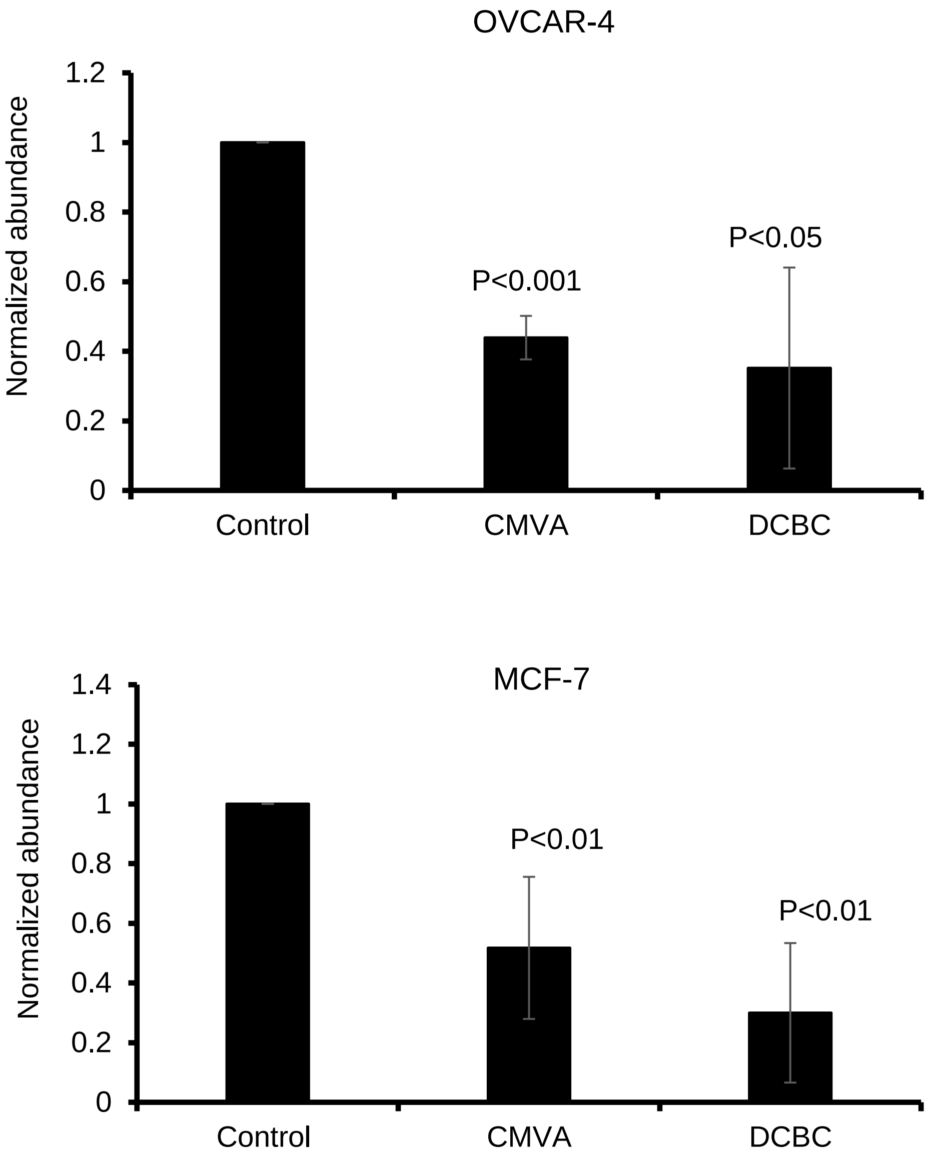

Supplementary Figure S6 Quantification of the effects of BCKDKi on Myc expression. OVCAR-4 OR MCF-7 cells were synchronized with nocodazole as described in the methods, treated with DMSO or 100 µM CMVA or DCBC for 72h, lysed and analysed by immunoblotting as shown. The results were quantified and normalized to Histone H3 expression.

S7 Effect of BCKDKi and paclitaxel in MDA-MB-468 cells

A.

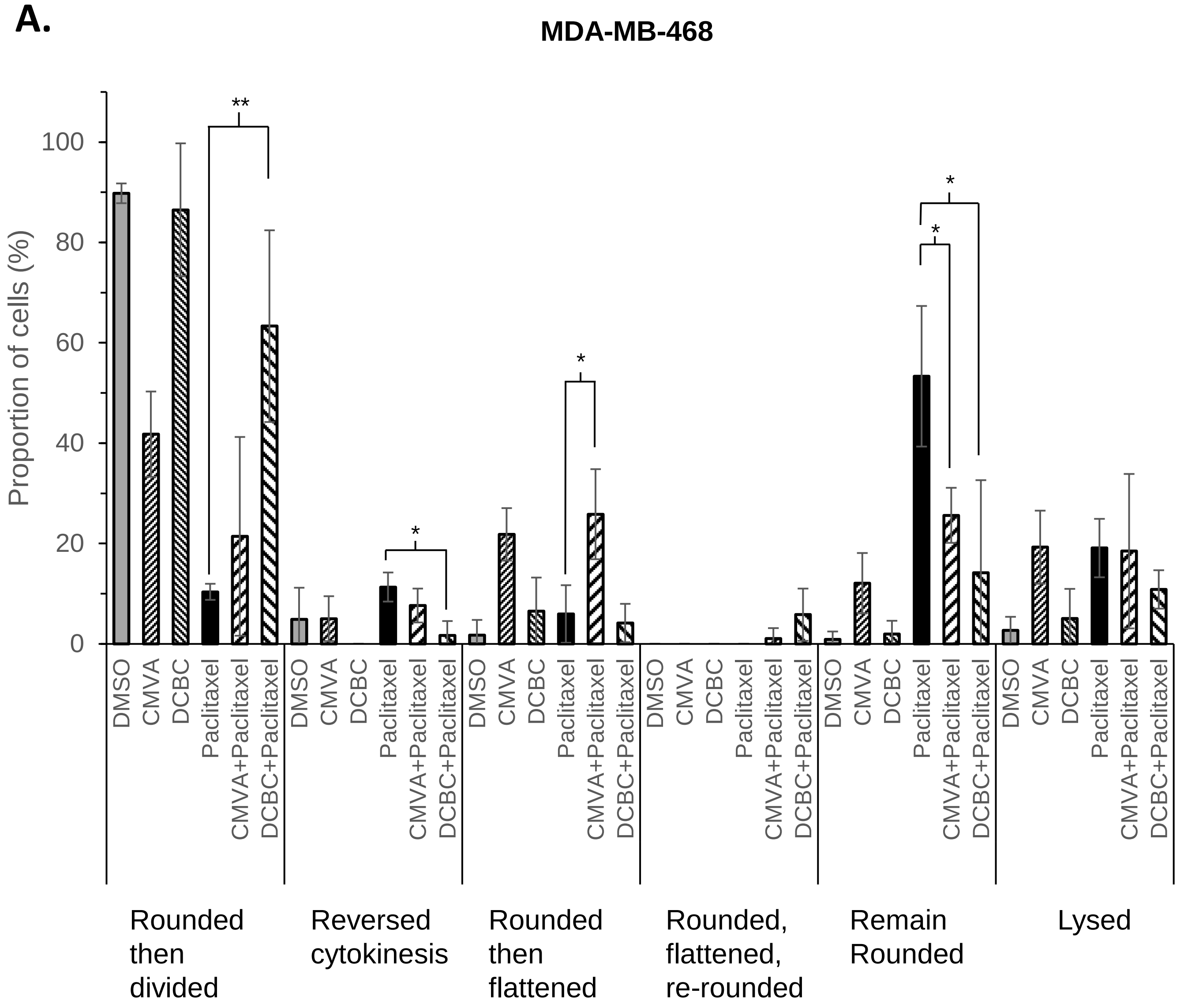

B.

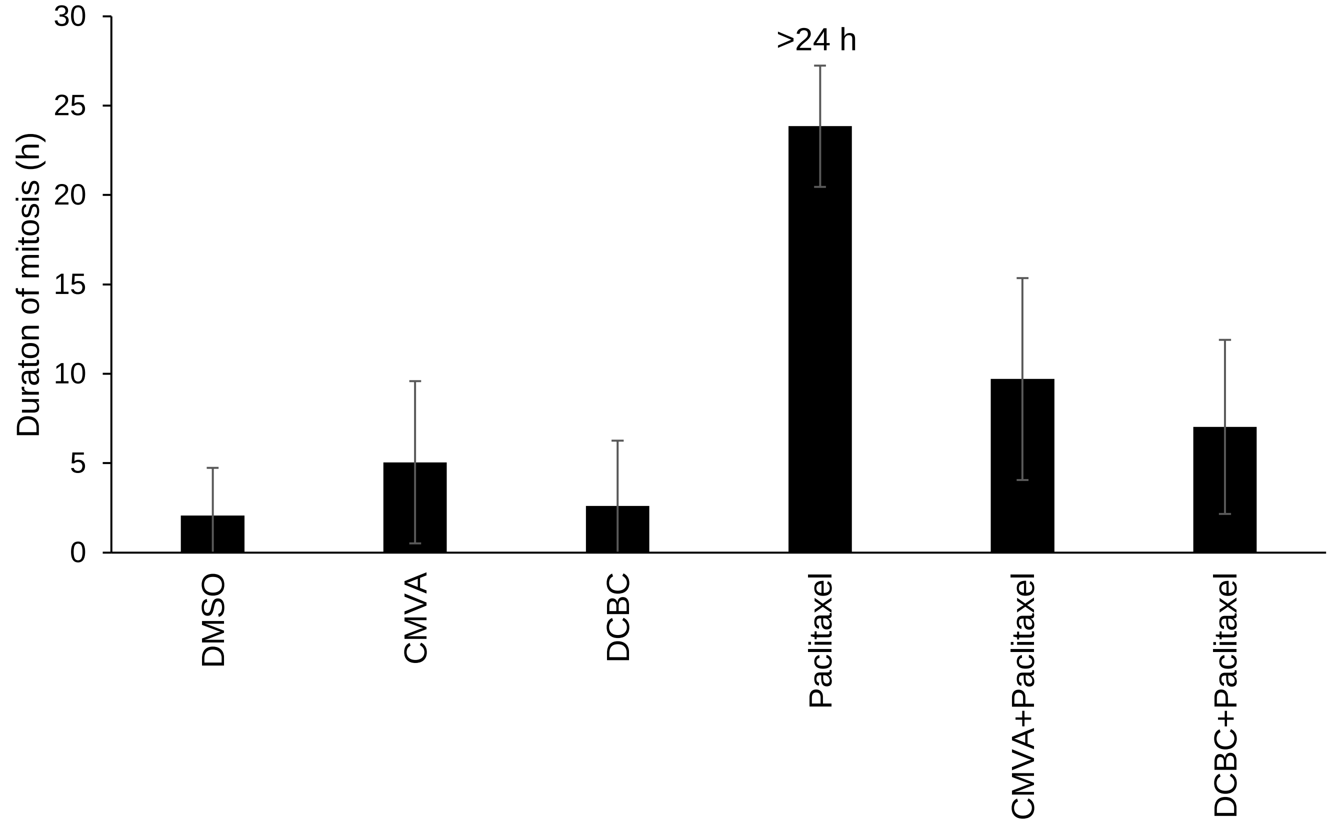

Supplementary Fig S7. Effect of BCKDK inhibitors on M-phase arrest induced by paclitaxel. A, MDA-MB-4868 cells were exposed to the indicated combinations of paclitaxel (9.5 nM), CMVA (100 µM) or DCBC (100 µM) and monitored by time-lapse microscopy. The time at which the cells rounded and their subsequent behaviour was classified (mean ± S.D., n=3 total of 100 cells counted for each condition) as "reversed cytokinesis" (cells started to divide, but then aborted division), "rounded then flattened" (cells did not divide but instead reverted to a flat morphology) or "rounded, flattened then re-rounded (as the previous group, but subsequently rounded again) or "remained rounded" (the cells remained rounded until the end of the recording period). Some cells fragmented, consistent with apoptosis and this is here recorded as "lysis" due to the lack of additional confirmatory evidence. Cell fate maps are shown for each cell in Fig S13-18. **B.** The time at which cells rounded was noted and time to flatten again determined and the interval this encompassed taken to represent the time required to complete mitosis. Cells treated with paclitaxel remained rounded throughout the 24h duration of the video and hence are labelled ">24 h".

S8

MCF7 cell fate
Treatment: DMSO

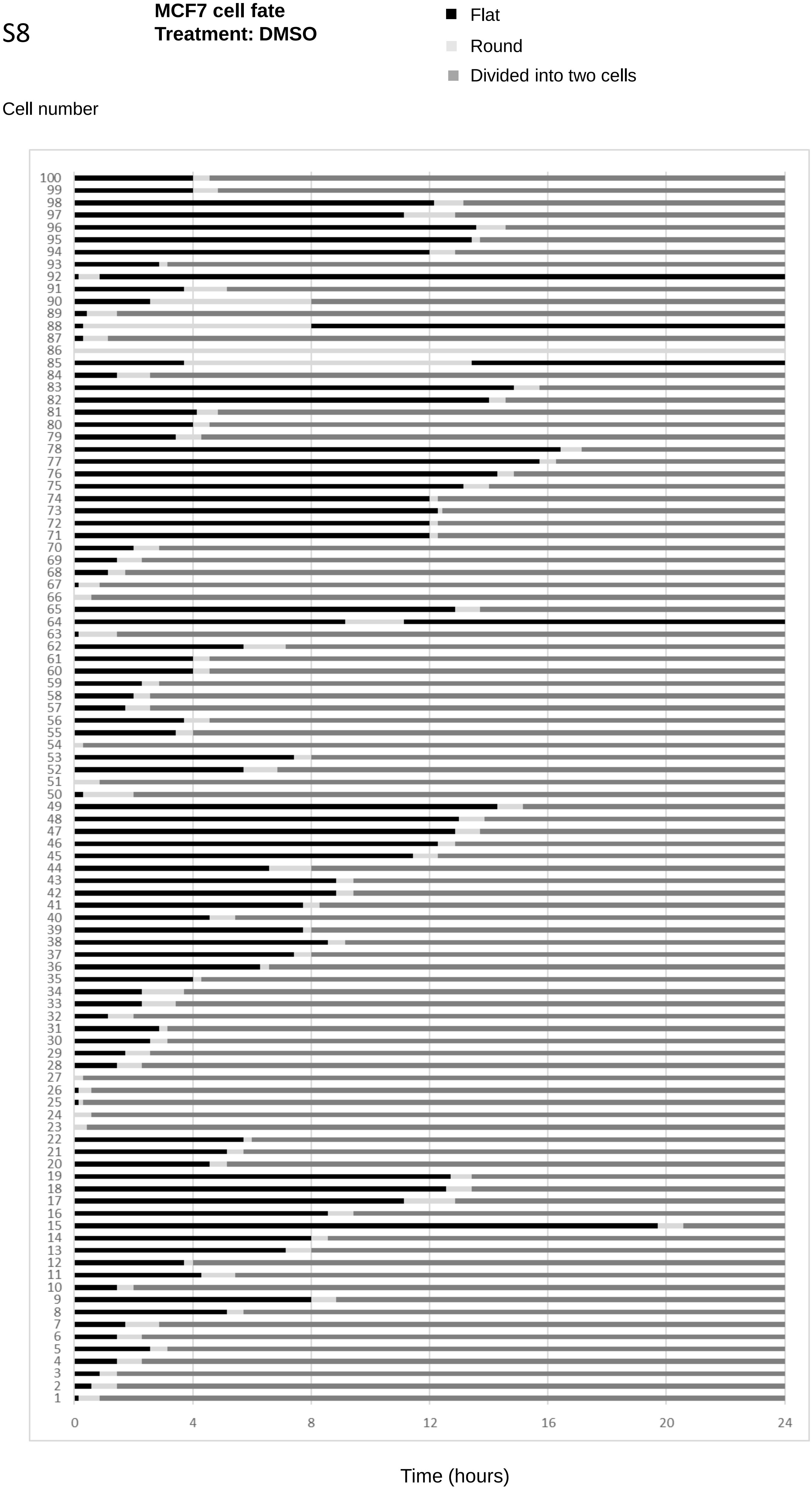

S9

MCF7 cell fate
Treatment: CMVA

Flat
Round
Divided into two cells
Reversed cytokinesis

Cell number

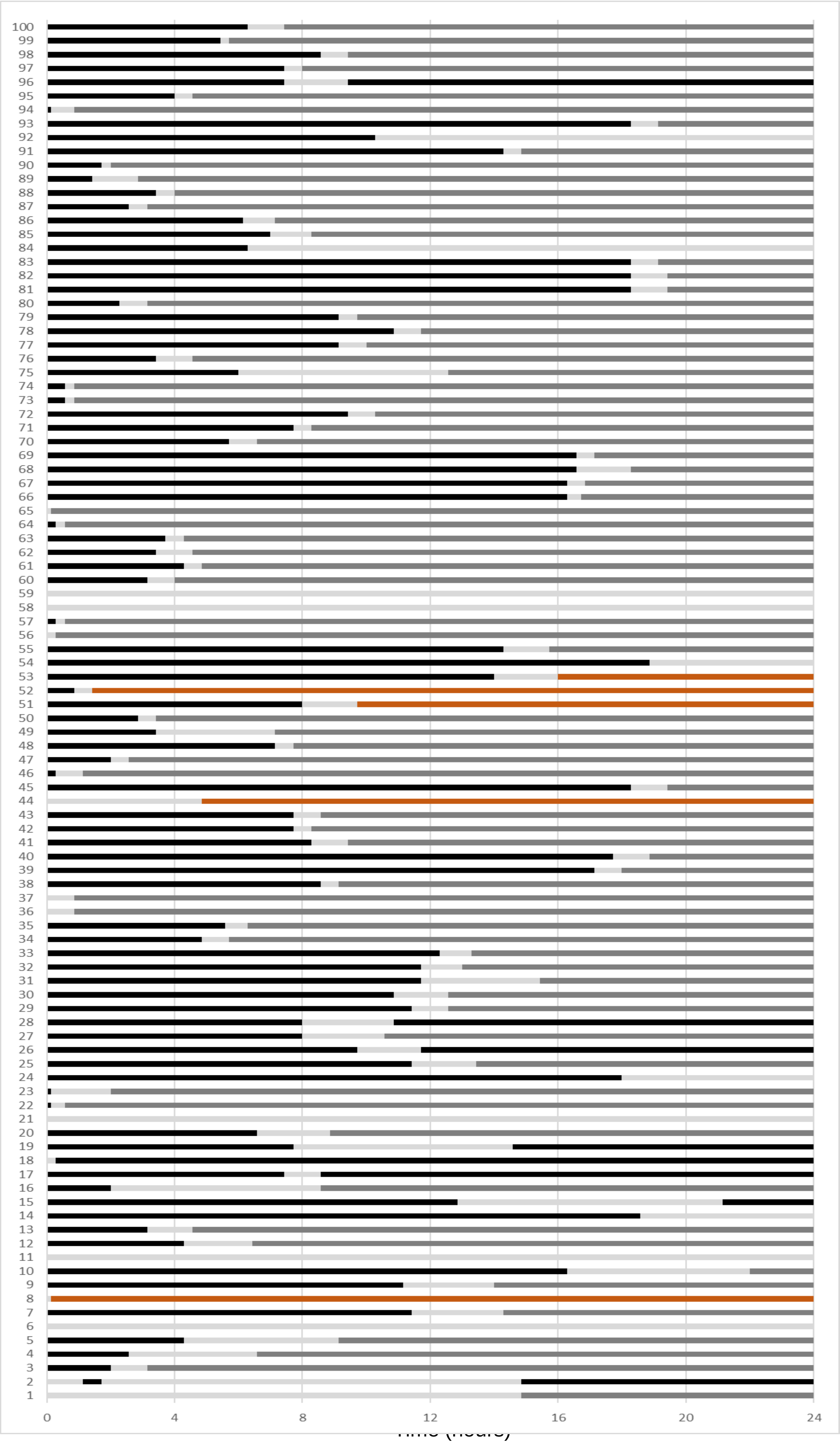

S10

MCF7 cell fate
Treatment: DCBC

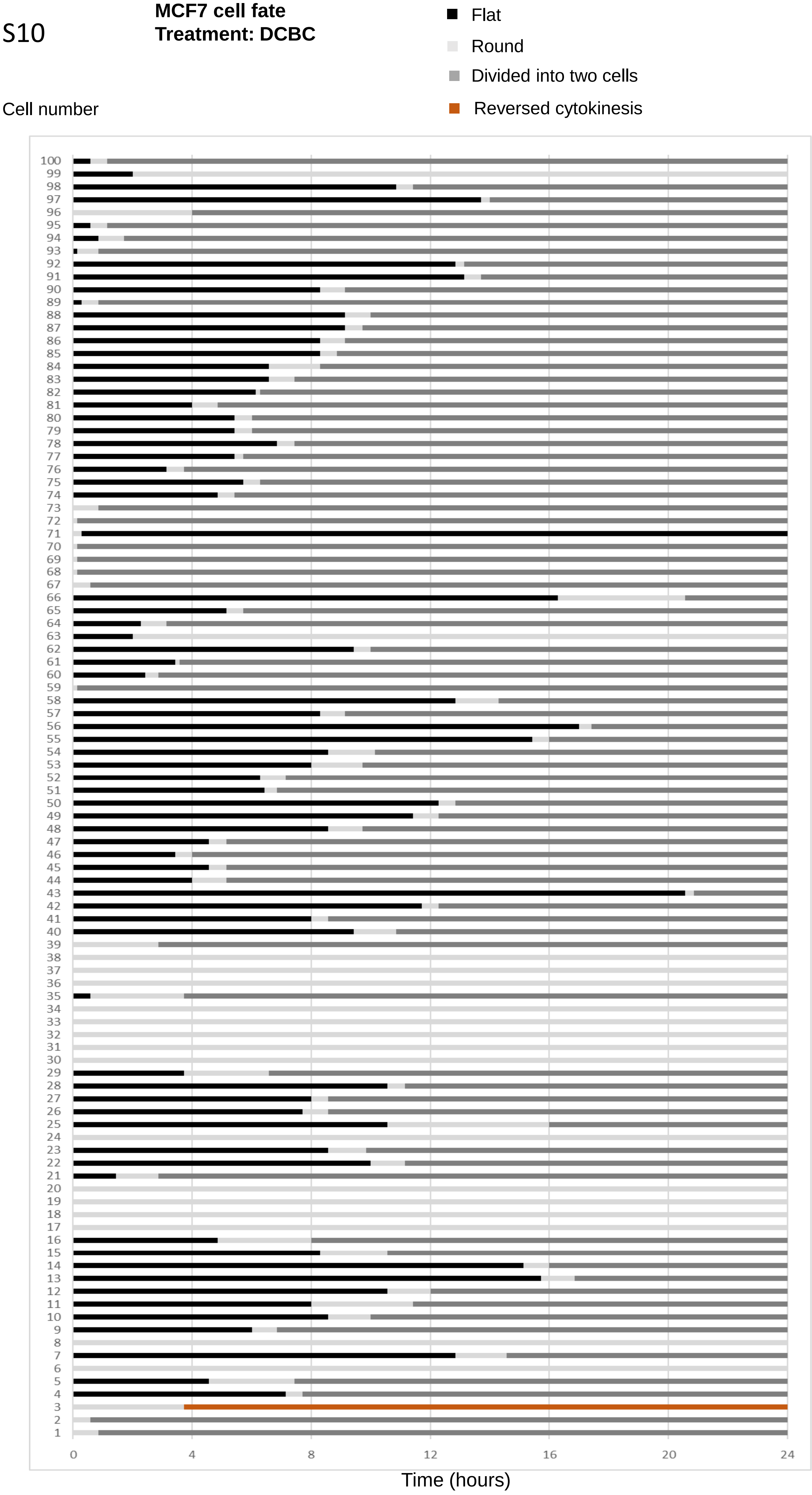

S11

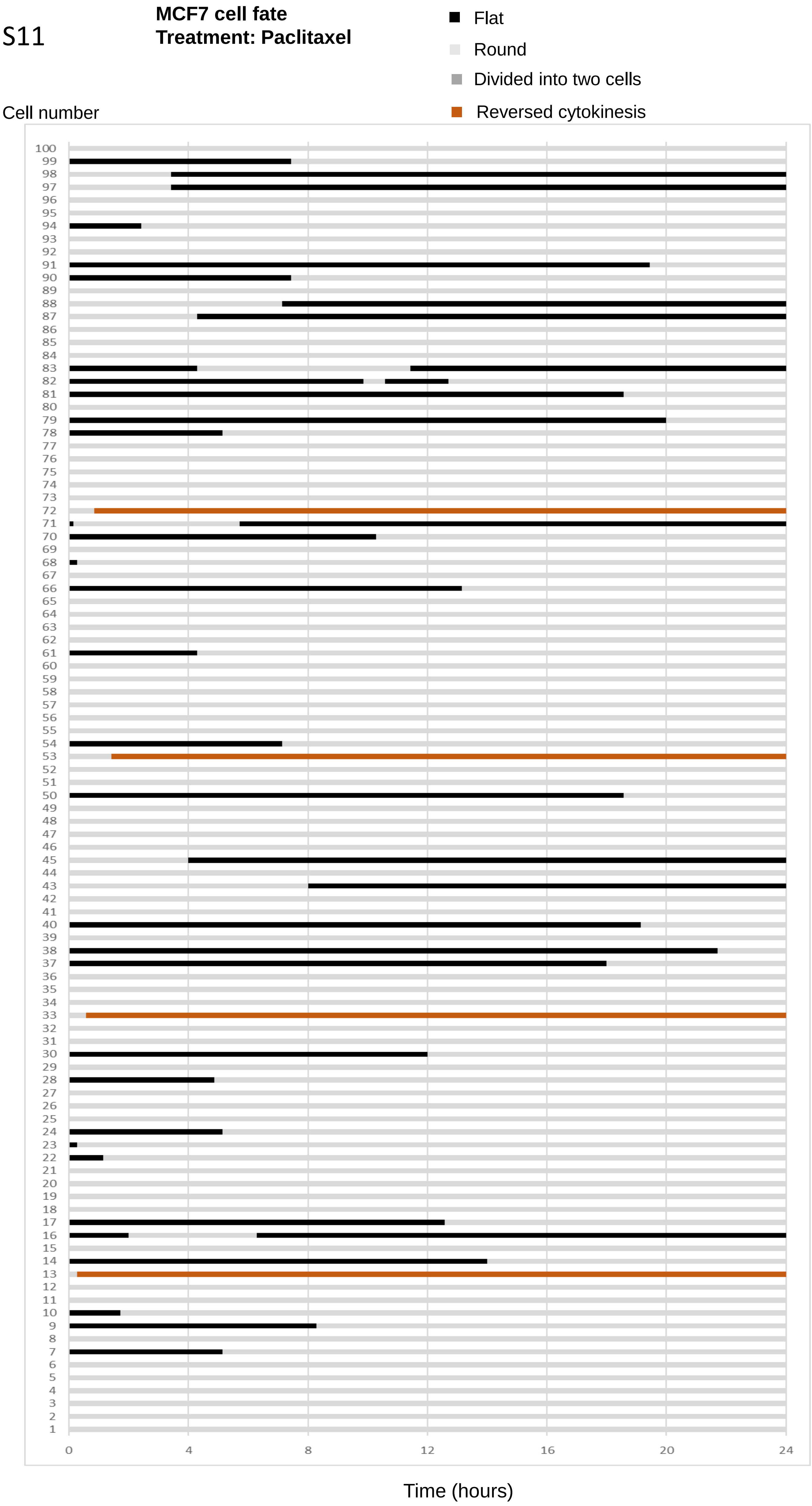

MCF7 cell fate
Treatment: Paclitaxel
Flat
Round
Divided into two cells
Reversed cytokinesis
Cell number
100
99
98
97
96
95
94
93
92
91
90
89
88
87
86
85
84
83
82
81
80
79
78
77
76
75
74
73
72
71
70
69
68
67
66
65
64
63
62
61
60
59
58
57
56
55
54
53
52
51
50
49
48
47
46
45
44
43
42
41
40
39
38
37
36
35
34
33
32
31
30
29
28
27
26
25
24
23
22
21
20
19
18
17
16
15
14
13
12
11
10
9
8
7
6
5
4
3
2
1
0
4
8
12
16
20
24
Time (hours)

S12

MCF7 cell fate
Treatment: Paclitaxel & CMVA

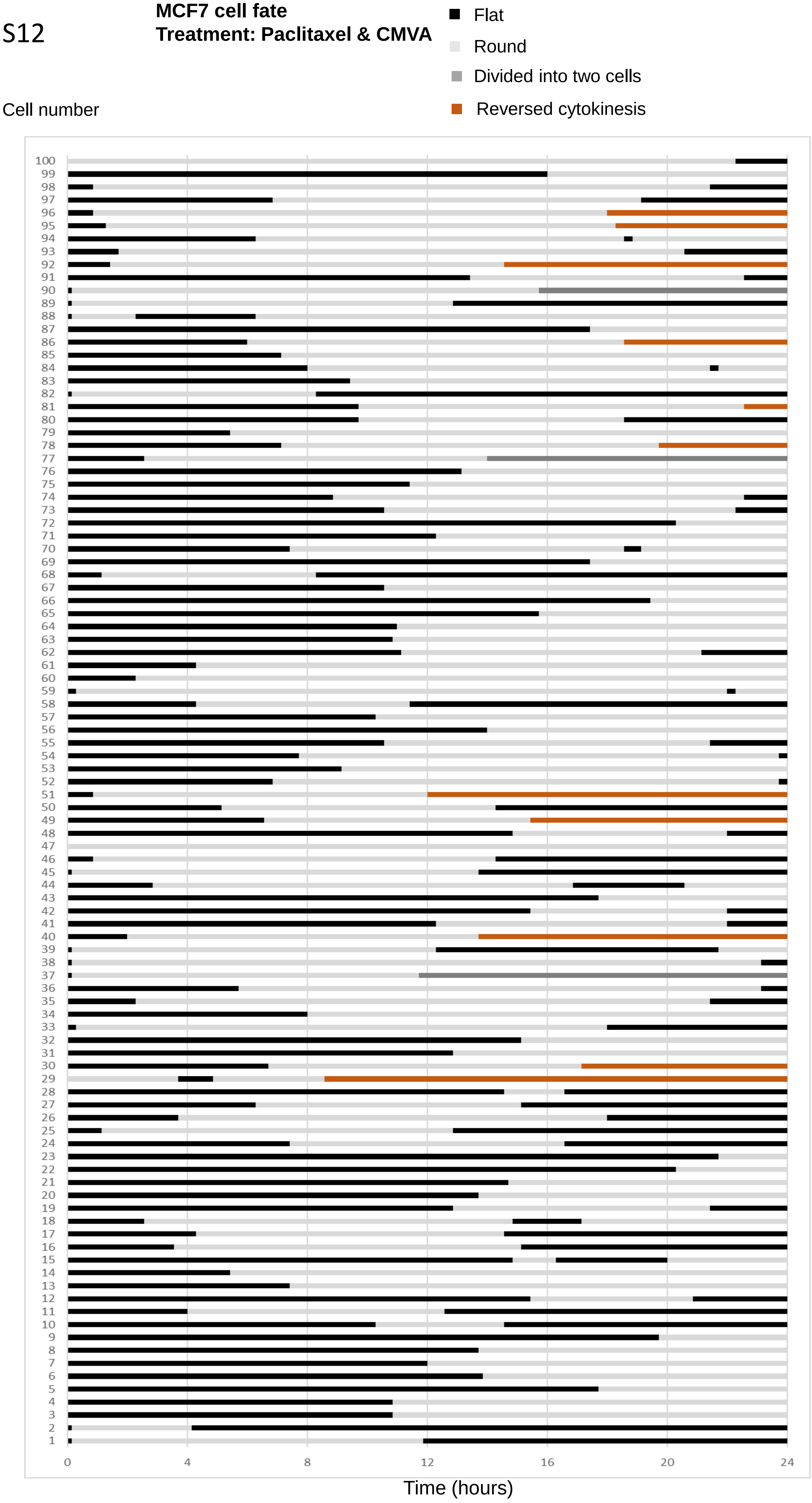

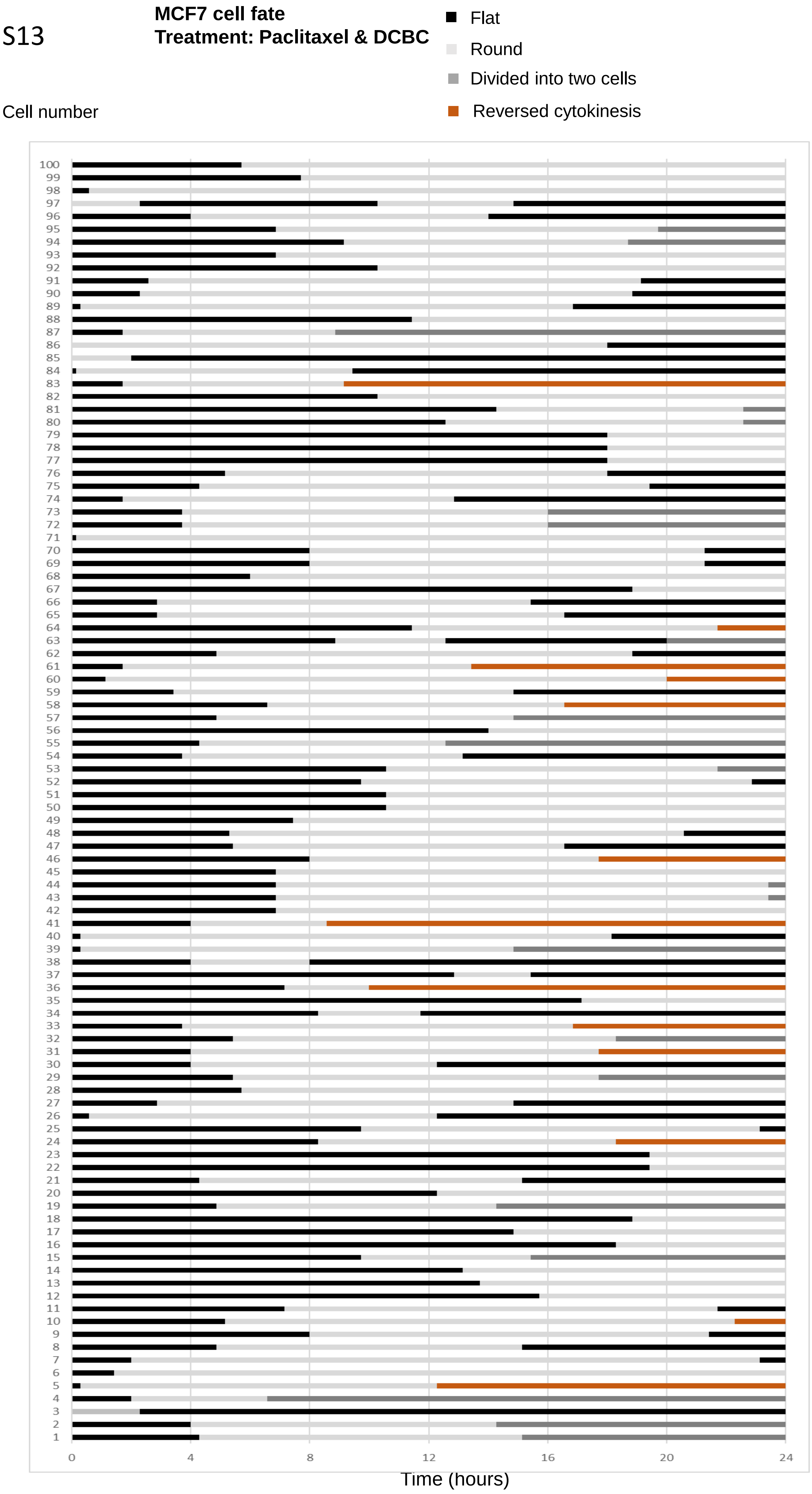

S13
MCF7 cell fate
Treatment: Paclitaxel & DCBC
Flat
Round
Divided into two cells
Reversed cytokinesis
Cell number
100
99
98
97
96
95
94
93
92
91
90
89
88
87
86
85
84
83
82
81
80
79
78
77
76
75
74
73
72
71
70
69
68
67
66
65
64
63
62
61
60
59
58
57
56
55
54
53
52
51
50
49
48
47
46
45
44
43
42
41
40
39
38
37
36
35
34
33
32
31
30
29
28
27
26
25
24
23
22
21
20
19
18
17
16
15
14
13
12
11
10
9
8
7
6
5
4
3
2
1
0
4
8
12
16
20
24
Time (hours)

S14

MDA-MB-468 cell fate
Treatment: DMSO

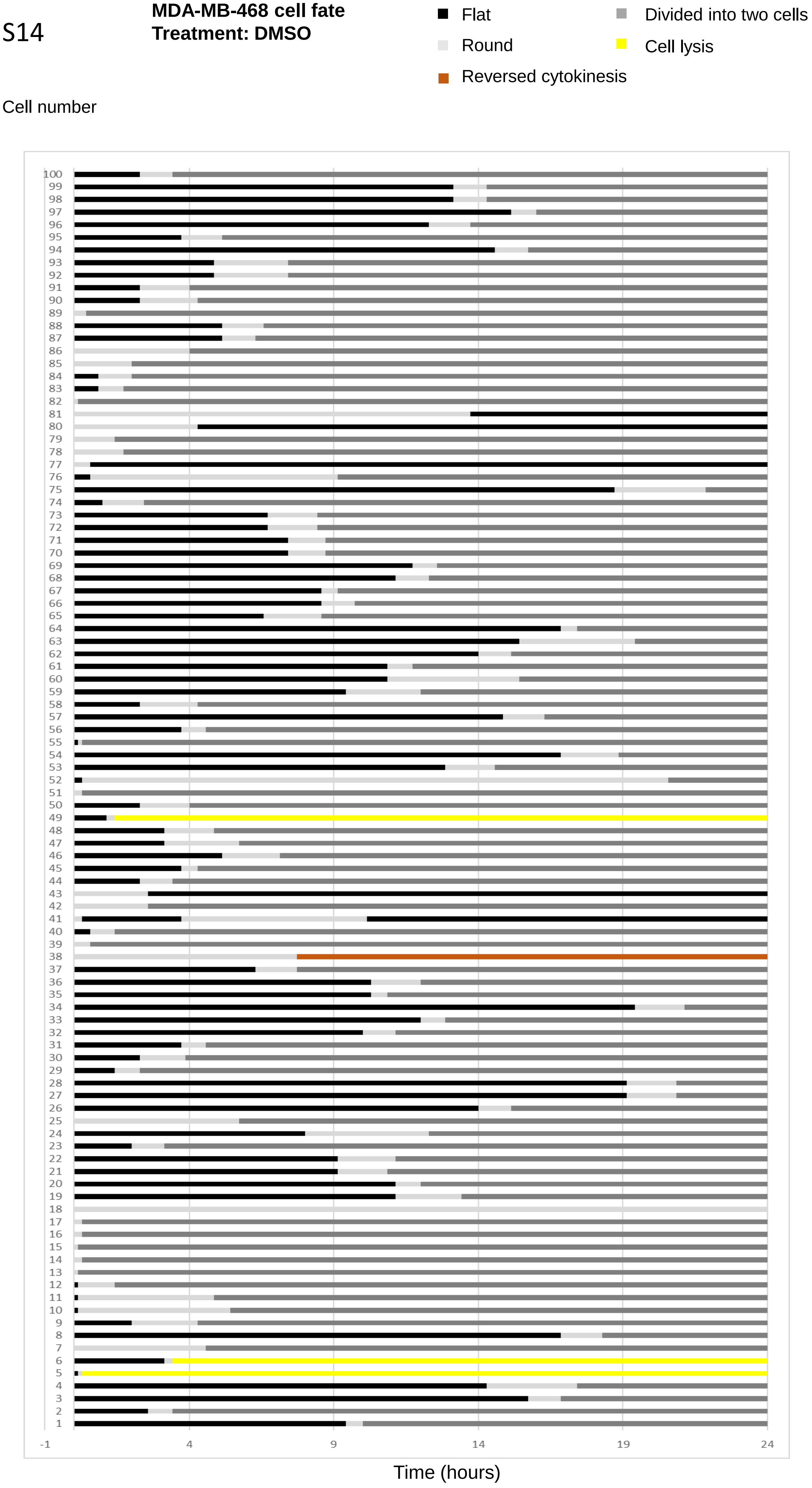

S15

MDA-MB-468 cell fate
Treatment: CMVA

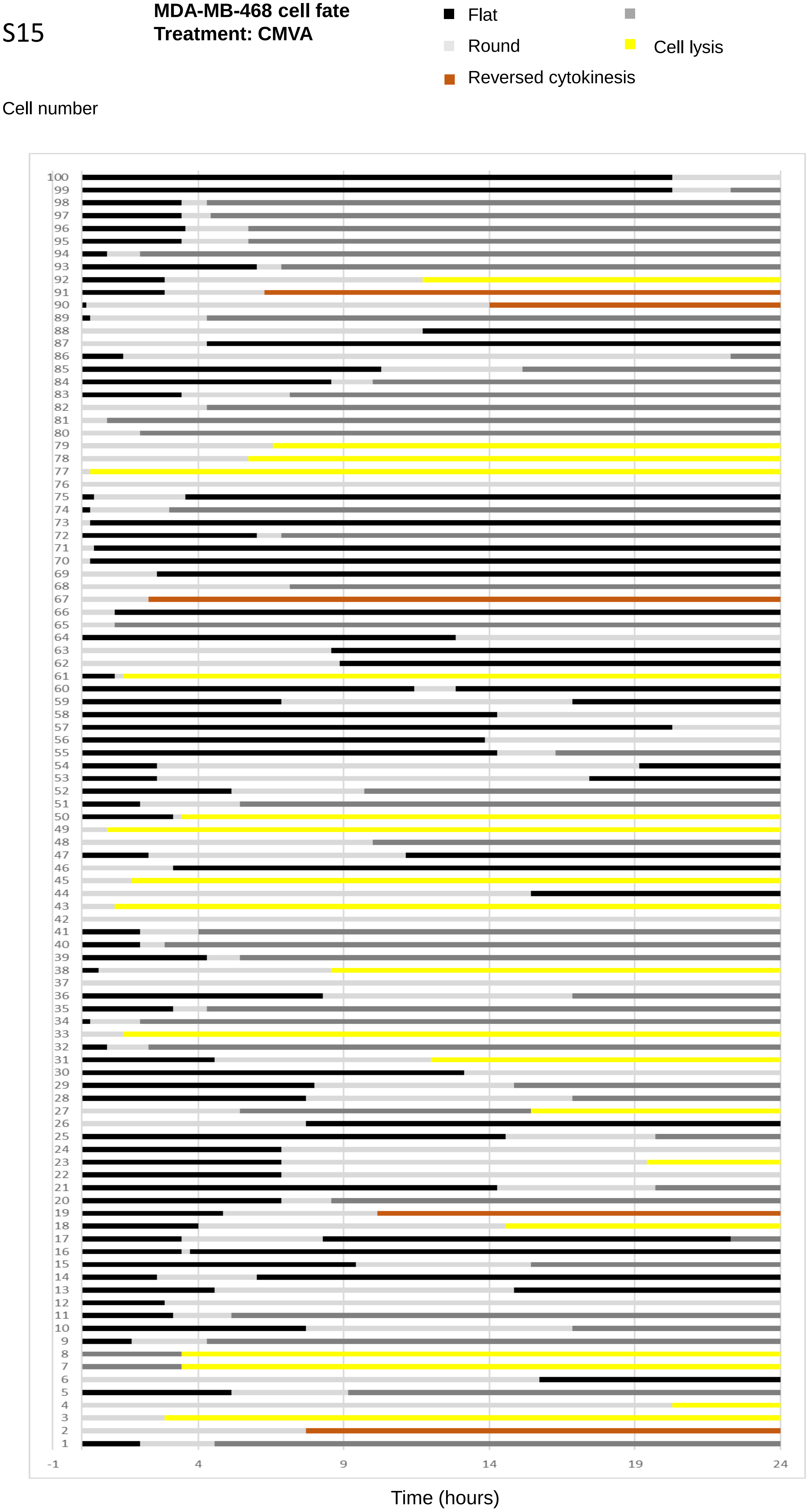

S16

MDA-MB-468 cell fate
Treatment: DCBC

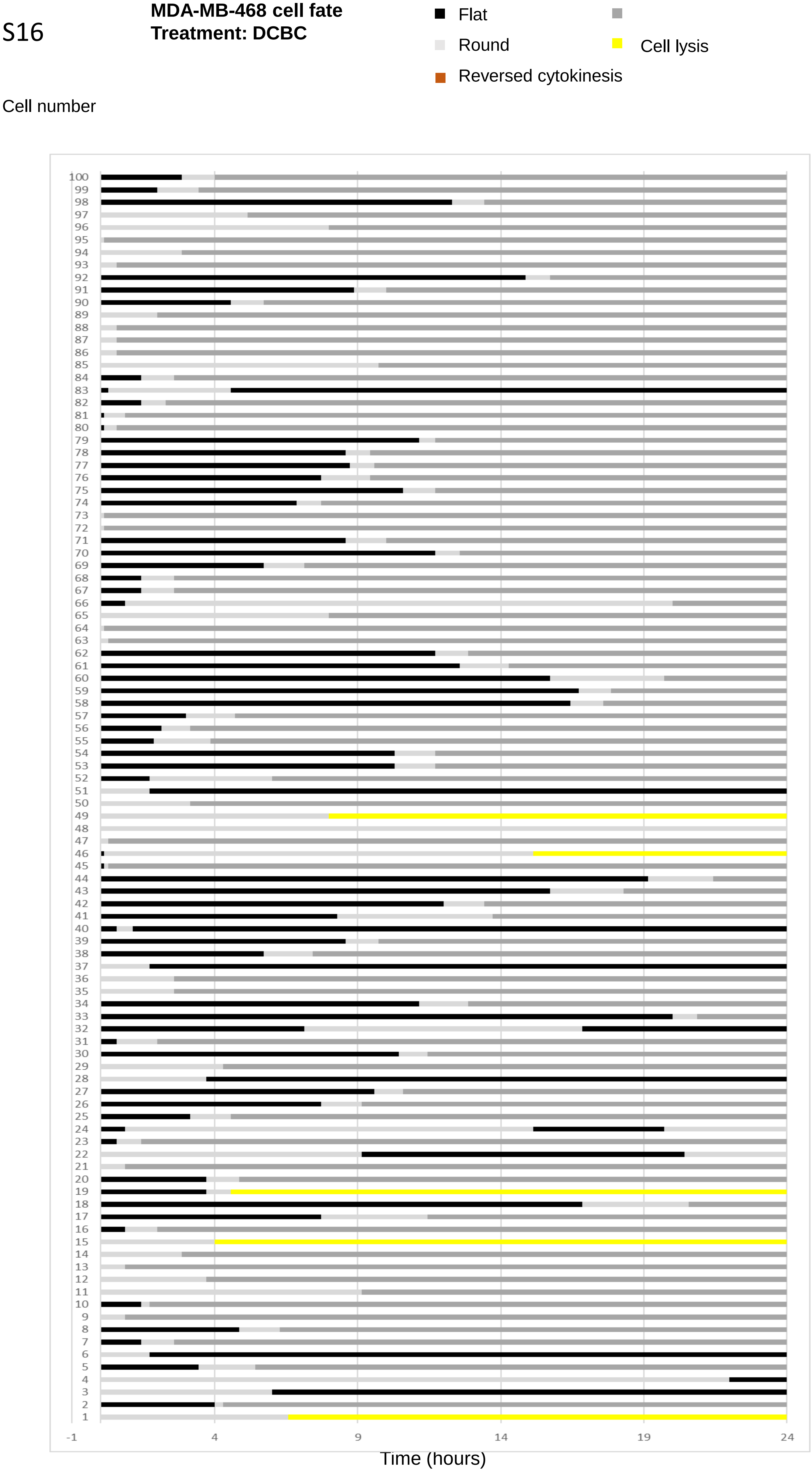

S17

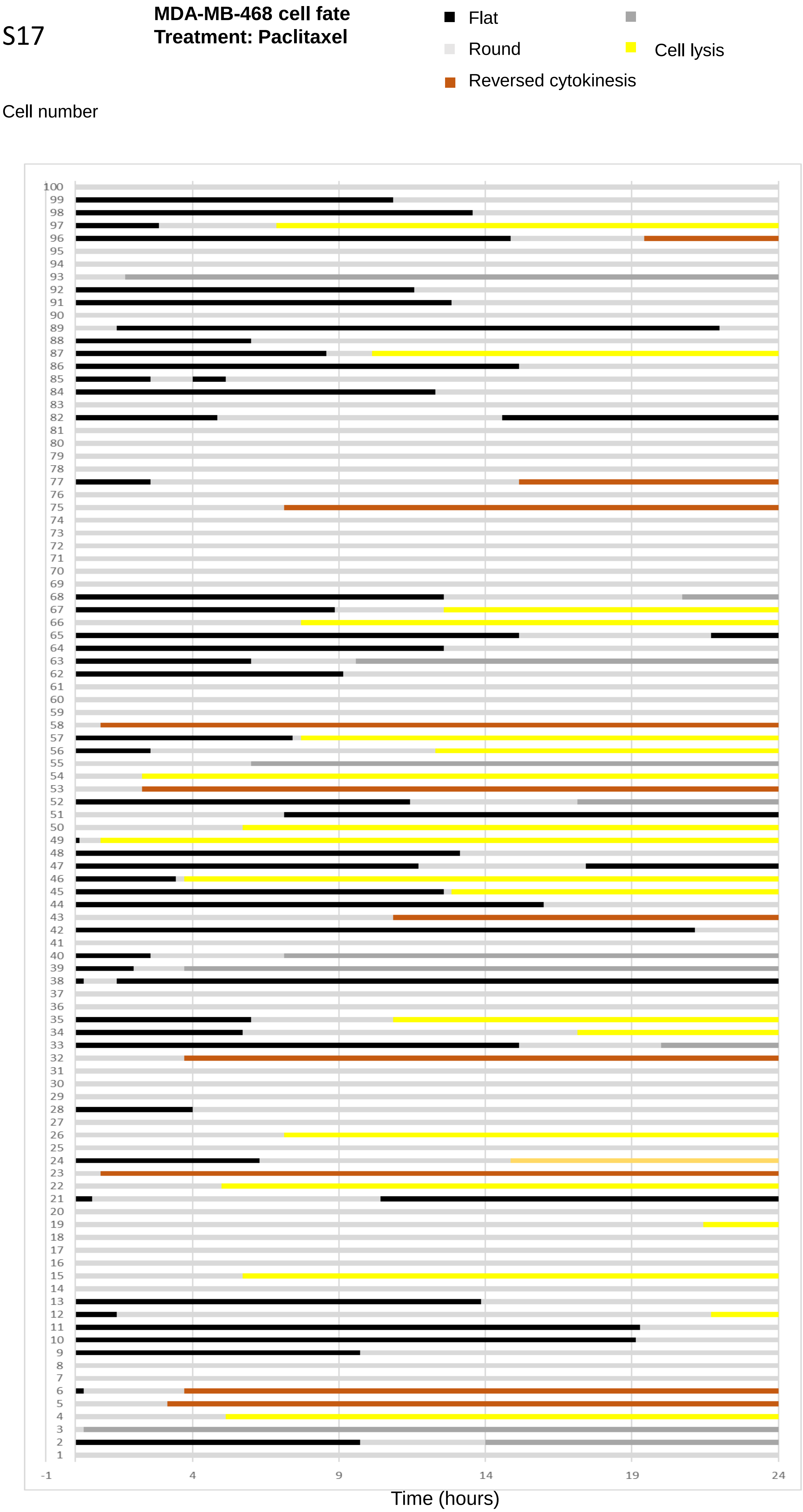
MDA-MB-468 cell fate
Treatment: Paclitaxel
Flat
Round
Reversed cytokinesis
Cell lysis
Cell number
Time (hours)

S18

MDA-MB-468 cell fate
Treatment: Paclitaxel & CMVA

Flat
Round
Reversed cytokinesis
Cell lysis

Cell number

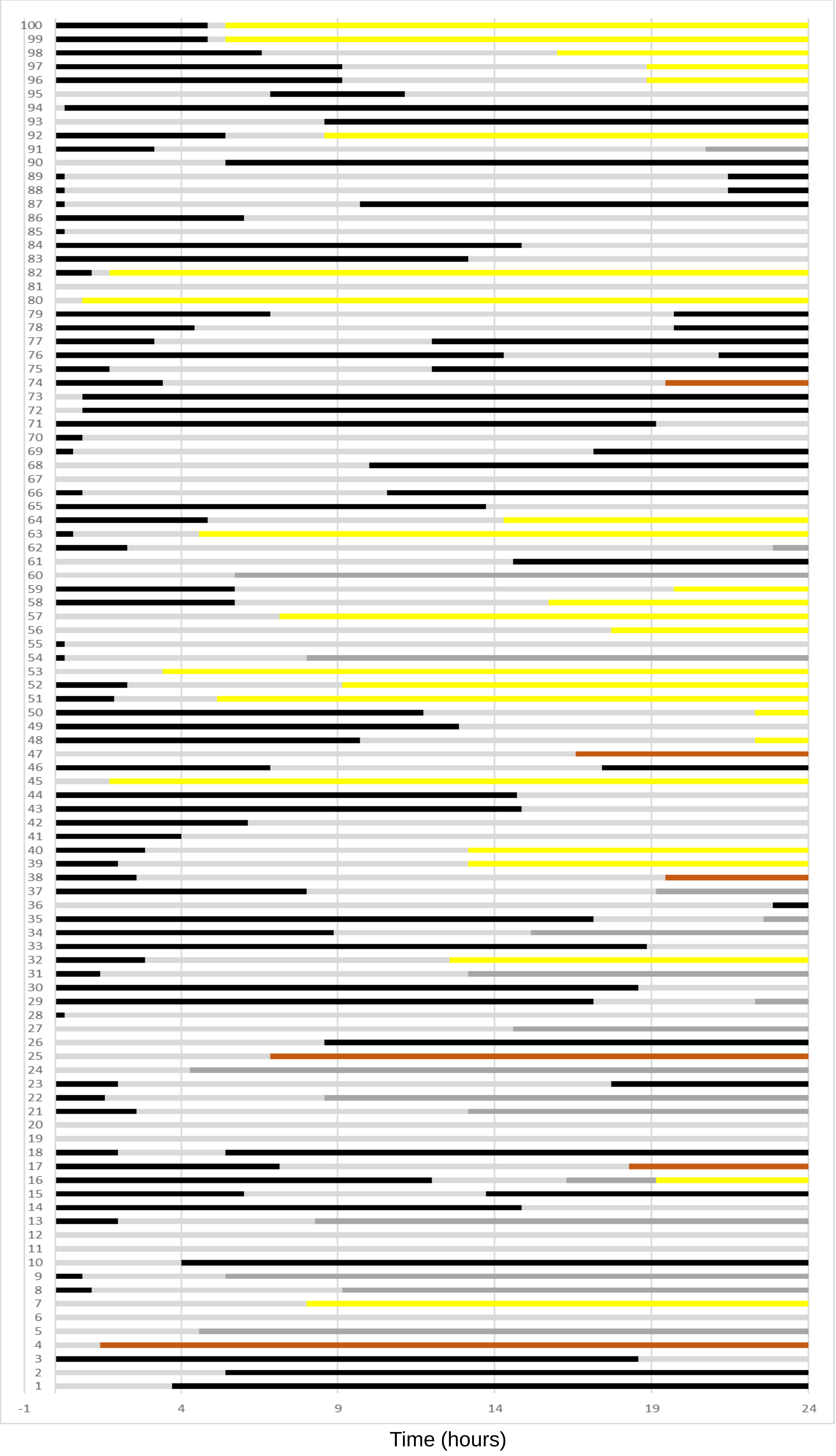

S19

MDA-MB-468 cell fate
Treatment: DCBC & paclitaxel

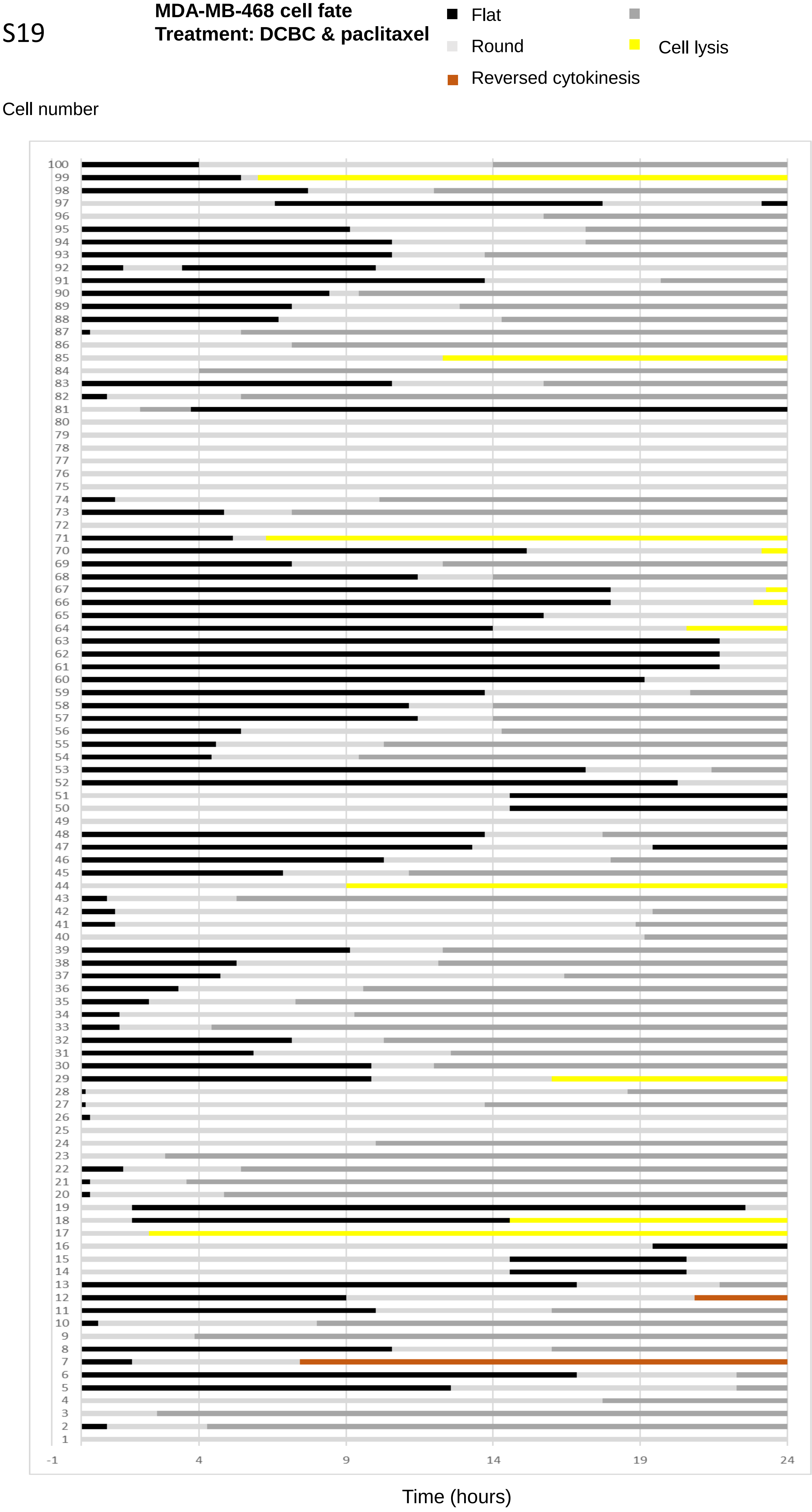

S20: All MCF7 Fate maps

DMSO

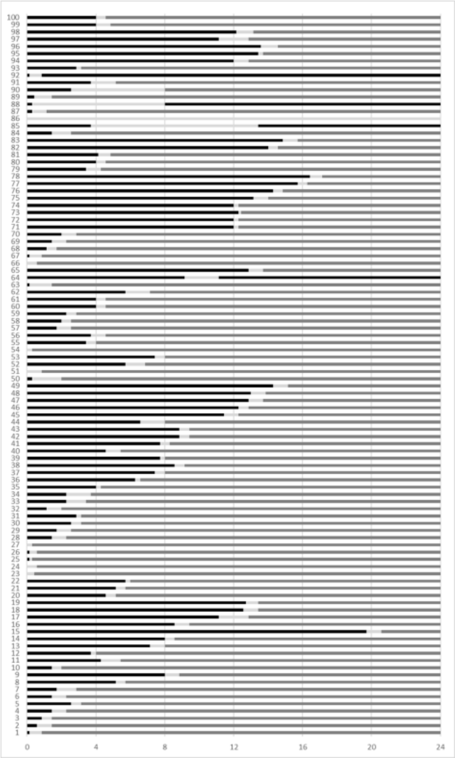

CMVA

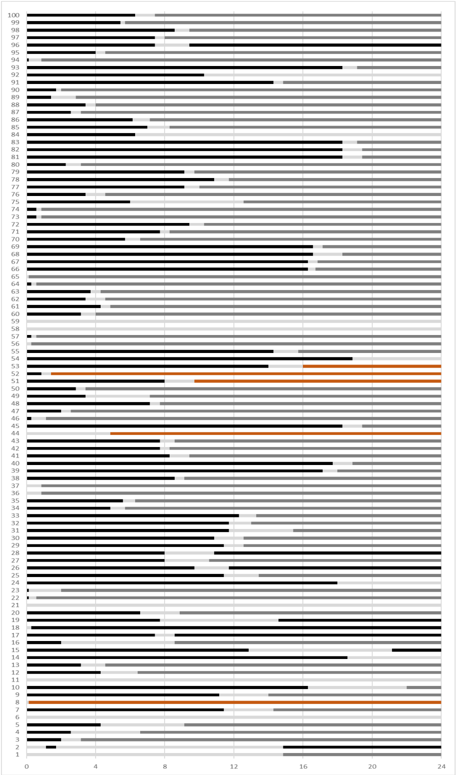

DCBC

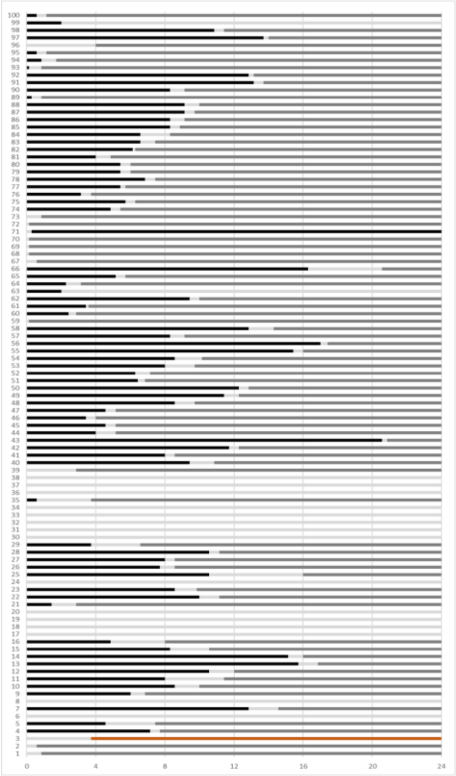

Paclitaxel

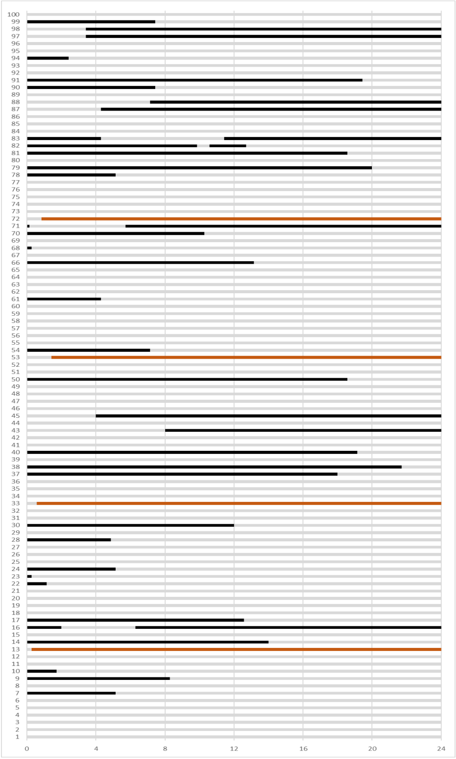

CMVA & Paclitaxel

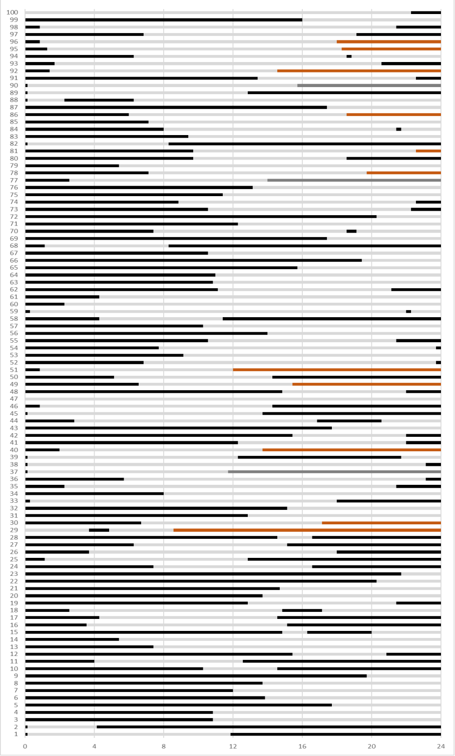

DCBC & Paclitaxel

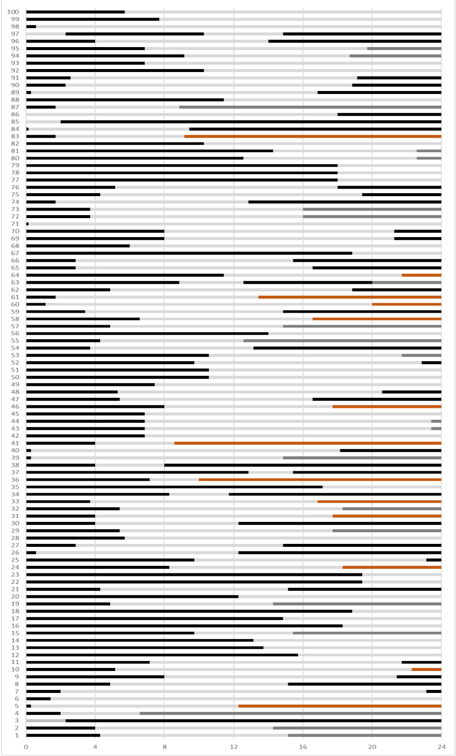

S20: All MDA-MB-468 fate maps

DMSO

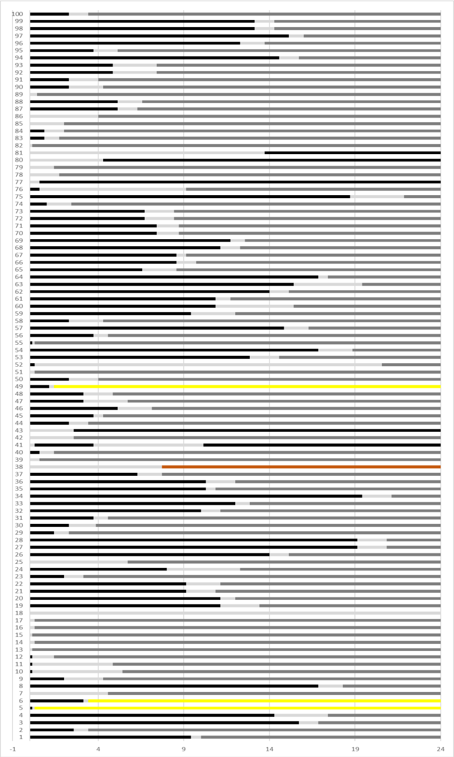

CMVA

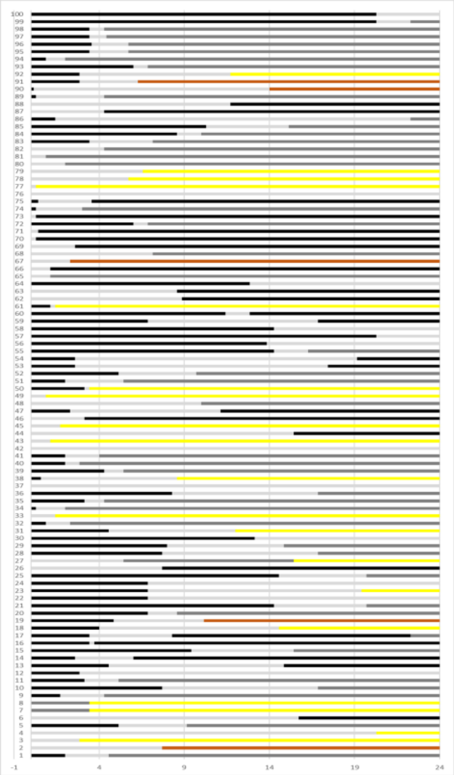

DCBC

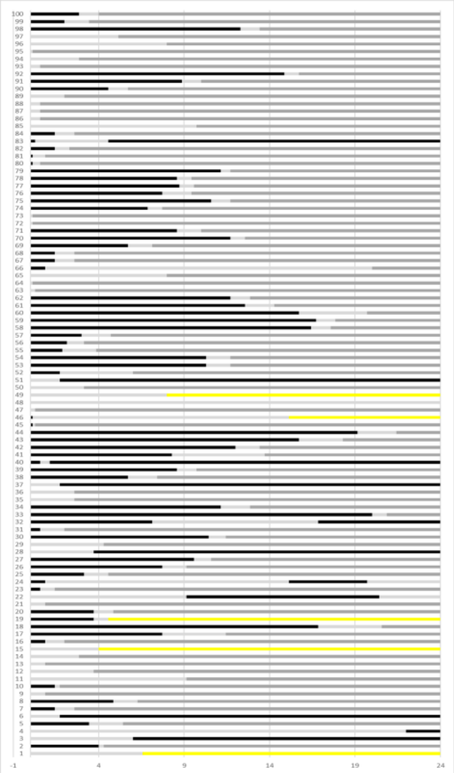

Paclitaxel

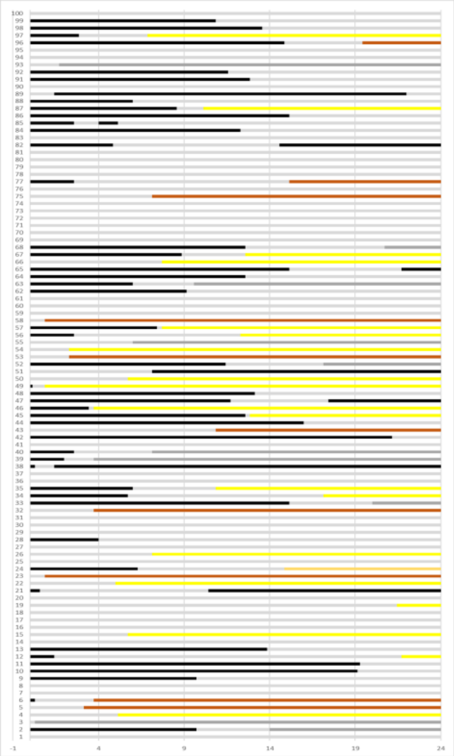

CMVA & Paclitaxel

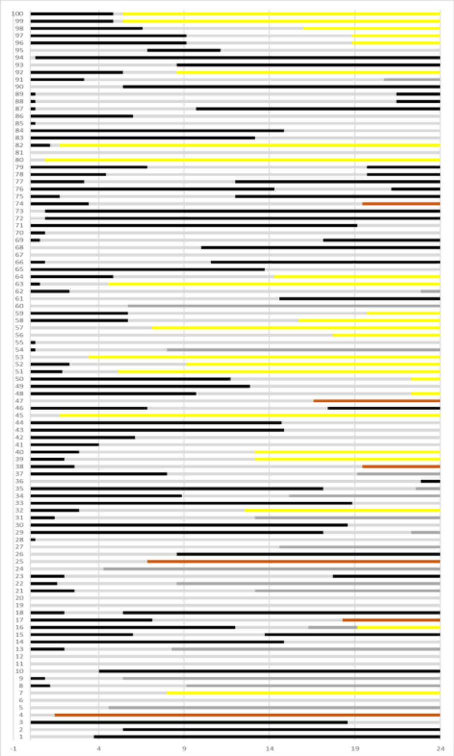

DCBC & Paclitaxel

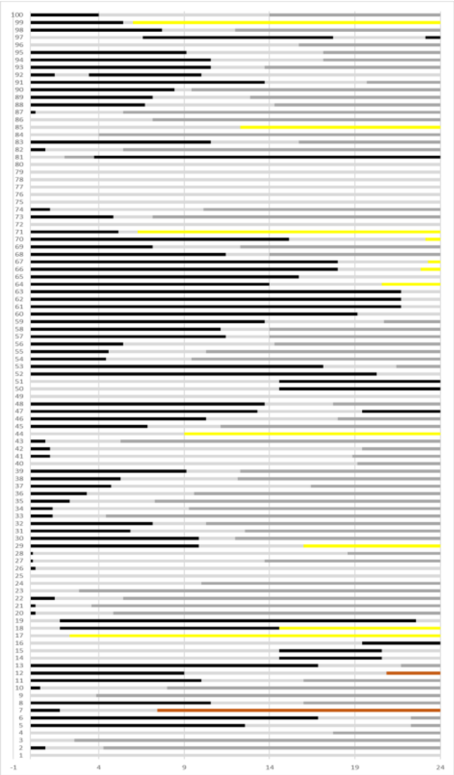

Supplementary Fig 8-21. Cell fate maps for cells exposed to combinations of paclitaxel and BCKDKi. MCF-7 (S7-12) or MDA-MB-468 (S13-18) cells were exposed to the indicated combinations of paclitaxel (9 nM), CMVA (100 µM) or DCBC (100 µM) and monitored by time-lapse microscopy. The time at which the cells rounded and their subsequent behaviour was recorded. Figure 20 (MCF-7) and figure 21 (MDA-MB-468) show a composite of the previous figures, to facilitate comparison.

S22 Cell cycle analysis of cells treated with paclitaxel and BCKDKi

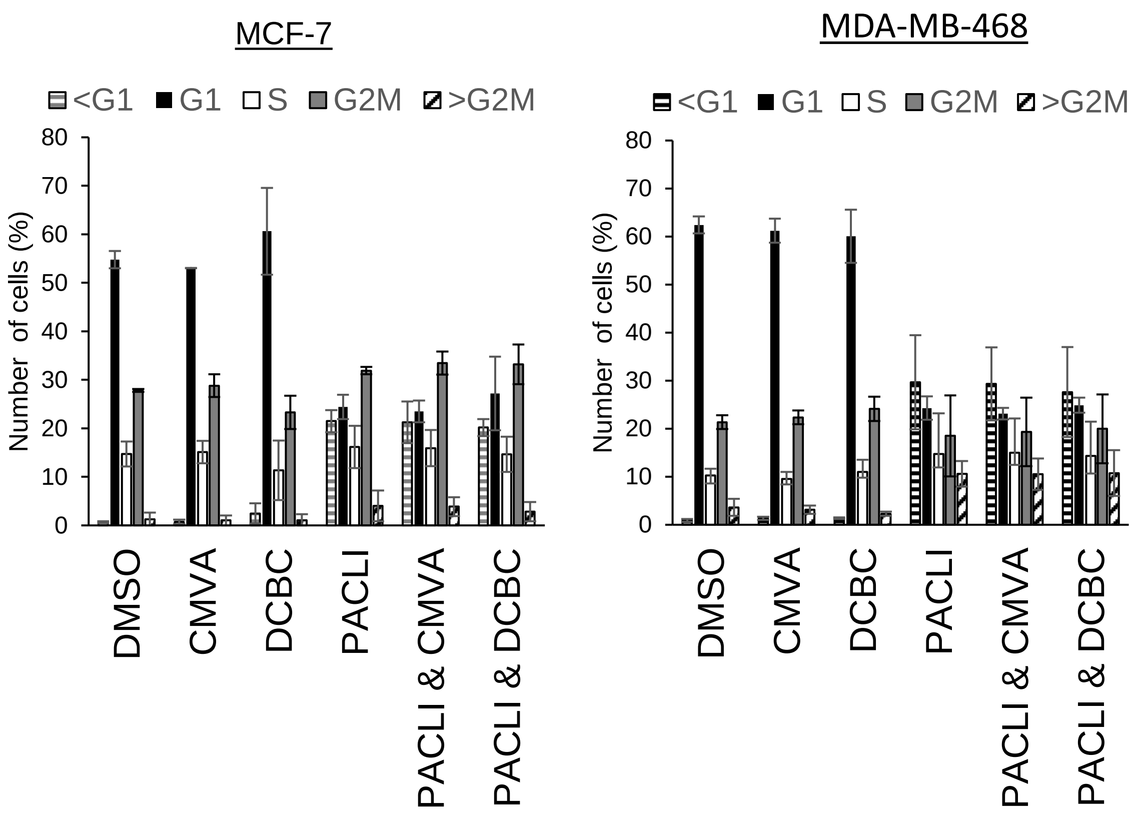

Supplementary figure 22 Cell cycle analysis of cells treated with the drug combination.

MCF-7 or MDA-MA-468 cells were exposed to DMSO, paclitaxel (9.5 nM) and/or CMVA (100 µM) or DCBC (100 µM). After 48 hours, the cells were collected, stained with propidium iodide and analysed by flow cytometry (mean ± S.D. n = 3). Inclusion of the BCKDK inhibitors with paclitaxel did not significantly increase the number of cells with greater than 4N content compared to cells exposed to paclitaxel alone.

Supplementary videos 1-12.

The videos show MCF7 (videos 1-5) or MDA-MB-468 cells (videos 6-12) treated with either paclitaxel alone (video 1 & 6) or paclitaxel and DCBC (remaining videos). The videos exemplify different types of cell behaviour after exposure to the drugs including mitotic arrest after exposure to paclitaxel (videos 1 and 6), mitosis (videos 3 and 8), cells rounding then re-flattening without dividing (videos 4 and 9,10), starting cytokinesis then reverting to a single cell ("reversing cytokinesis", video 5 and 11) and cell fragmenting ("lysis", video 12). One second of the video represents 8.5 minutes of real time.
